# Insights into the SARS-CoV-2 ORF6 Mechanism of Action

**DOI:** 10.3390/ijms241411589

**Published:** 2023-07-18

**Authors:** Elena Krachmarova, Peicho Petkov, Elena Lilkova, Nevena Ilieva, Miroslav Rangelov, Nadezhda Todorova, Kristina Malinova, Rossitsa Hristova, Genoveva Nacheva, Anastas Gospodinov, Leandar Litov

**Affiliations:** 1 Institute of Molecular Biology “Acad. Roumen Tsanev”, Bulgarian Academy of Sciences, 1113 Sofia, Bulgaria; elenakrachmarova@bio21.bas.bg (E.K.); kristina.malinova94@abv.bg (K.M.); hristova_r@bio21.bas.bg (R.H.); genoveva@bio21.bas.bg (G.N.); 2 Faculty of Physics, Sofia University “St. Kliment Ohridski”, 1164 Sofia, Bulgaria; leandar.litov@cern.ch; 3 Institute of Information and Communication Technologies, Bulgarian Academy of Sciences, 1113 Sofia, Bulgaria; elena.lilkova@iict.bas.bg (E.L.); nevena.ilieva@iict.bas.bg (N.I.); 4 Institute of Organic Chemistry with Centre of Phytochemistry, Bulgarian Academy of Sciences, 1113 Sofia, Bulgaria; marangelov@gmail.com; 5 Institute of Biodiversity and Ecosystem Research, Bulgarian Academy of Sciences, 1113 Sofia, Bulgaria; nadeshda@abv.bg

**Keywords:** SARS-CoV-2 ORF6–RAE1 binding, 3D molecular structure modelling, molecular dynamics simulations, cell cycle, cyclin E, R-loops, DNA replication stress

## Abstract

ORF6 is responsible for suppressing the immune response of cells infected by the SARS-CoV-2 virus. It is also the most toxic protein of SARS-CoV-2, and its actions are associated with the viral pathogenicity. Here, we study in silico and in vitro the structure of the protein, its interaction with RAE1 and the mechanism of action behind its high toxicity. We show both computationally and experimentally that SARS-CoV-2 ORF6, embedded in the cytoplasmic membranes, binds to RAE1 and sequesters it in the cytoplasm, thus depleting its availability in the nucleus and impairing nucleocytoplasmic mRNA transport. This negatively affects the cellular genome stability by compromising the cell cycle progression into the S-phase and by promoting the accumulation of RNA–DNA hybrids. Understanding the multiple ways in which ORF6 affects DNA replication may also have important implications for elucidating the pathogenicity of SARS-CoV-2 and developing therapeutic strategies to mitigate its deleterious effects on host cells.

## 1. Introduction

Nearly three years after its outbreak at the end of 2019, the unprecedented global COVID-19 pandemic is starting to subside due to the successful development of vaccines and antiviral treatments. Nonetheless, COVID-19 still poses an acute global health emergency due to its numerous long-lasting adverse health effects on the world’s population. The causative agent of the pandemic is the SARS-CoV-2 beta-coronavirus, which is an enveloped, positive sense, single-stranded RNA virus [1]. The genome of the virus is ~30 kb in size and encodes 29 proteins—4 structural, 16 nonstructural, and 9 accessory proteins [2]. While the latter are not strictly necessary for viral replication and assembly, they play a crucial role in immune evasion and viral pathogenicity [3,4].

Open reading frame 6 (ORF6) is an accessory protein uniquely expressed in the Sarbecovirus subgenus of Betacoronaviruses, to which the SARS-CoV and SARS-CoV-2 viruses belong [5]. ORF6 is translated from its own subgenomic RNA, transcribed by the replication–transcription complexes in virus-induced cytosolic double-membrane vesicles derived from endoplasmic reticulum membranes [6,7,8,9,10,11,12]. Its main function is to antagonize the host cell interferon signaling pathways, thus limiting the immune response to the infection [5,13,14,15,16]. SARS-CoV-2 has a high degree of similarity with SARS-CoV; however, a limited degree of similarity is observed in the accessory proteins, with ORF6 having the lowest homology of only 69% [1,17,18].

SARS-CoV-2 ORF6 is a polypeptide with 61 amino acids (aa) with an amphipathic N-terminal portion (aa 1–40) and a highly polar C terminus. It was shown that ORF6 is mainly localized in the endoplasmic reticulum (ER) and Golgi apparatus (GA) as well as autophagosome and lysosomal membranes [8,14,19,20,21,22,23]. Its N-terminal aa residues 2–37 form an α-helix inserted in the membrane [24,25]. Similar to the other ORF6 proteins, the function of SARS-CoV-2 ORF6 is to limit the immune response to the infection. This is achieved by two different mechanisms: on the one hand, it antagonizes STAT1 nuclear translocation [13,14,15], and on the other, it prevents mRNA transport via interaction with the nucleopore complex components nucleoporin 98 (NUP98) and ribonucleic acid export 1 (RAE1) [15,16,26,27]. However, the detailed mechanism of the pathogenic immune-suppressive action of ORF6 on the host target proteins is not yet well understood. Furthermore, ORF6 was shown to significantly contribute to the pathogenicity of the virus, having the highest cytotoxicity among the SARS-CoV-2 proteins [19]. All this prompts an active search for possible inhibitors of this protein. However, despite the significant research focus and efforts, ORF6 is one of the dozen proteins of SARS-CoV-2 whose 3D structures have not yet been resolved [2], substantially hindering the development of inhibitors.

In this study, we report results from both in silico and in vitro investigations on the SARS-CoV-2 ORF6 protein’s mechanism of action. Our data demonstrate that ORF6 accomplishes its effects by binding the RAE1 protein and sequestering it in the cytoplasm. We built a 3D model of the SARS-CoV-2 ORF6 protein inserted in an ER membrane and studied its interaction with RAE1 using molecular dynamics (MD) simulations. RAE1 binds one or more ORF6 molecules very stably, which immobilizes it on membrane organelles in the cytoplasm. As a consequence, the concentration of RAE1 in the cell’s nucleus is reduced significantly, strongly suppressing mRNA transport from the nucleus to the cytoplasm. These observations were confirmed experimentally by studying the co-localization of both proteins in ORF6-overexpressing and control cells. Further, we studied the impact of the SARS-CoV-2 ORF6 interaction with RAE1 on the host cell, specifically on DNA replication. The effects are manifested in at least two ways: by compromising cell progression into the S-phase and by promoting the accumulation of RNA–DNA hybrids, which are a major source of genome instability.

## 2. Results

### 2.1. Structure of ORF6 in Water Solvent

For computational studies of the ORF6–RAE1 interaction, the 3D structure of ORF6 is a prerequisite. The EXCALATE4COV input model of ORF6 is presented in Supplementary Figure S1. It contains four somewhat distorted helices (aa Leu${}^{4}$–Thr${}^{10}$, Leu${}^{16}$–Trp${}^{27}$, Asp${}^{30}$–Leu${}^{40}$ and Glu${}^{46}$–Glu${}^{54}$) connected by short loops (2–5 aa long). Starting from this structure, we performed an additional 7.6 µs of folding MD simulations. The secondary structure plot of the protein along the MD trajectory is shown in Supplementary Figure S2. The protein remained primarily helical. The distorted helices stabilized and transitioned to very well-defined α-helices, encompassing Leu${}^{4}$–Val${}^{9}$, Leu${}^{16}$–Val${}^{24}$, Thr${}^{31}$–Ser${}^{43}$ and Glu${}^{46}$–Glu${}^{55}$. The most important structural alteration was the change in the helices mutual orientation. The total protein solvent accessible surface area (SASA), as well as its decomposition in hydrophobic, polar and charged contributions, are presented in Supplementary Figure S3. After an initial jump, the total SASA of the protein dropped again to around the initial value and remained stable after the second microsecond. However, because of tertiary structure changes, the SASA of the hydrophobic aa residues decreased by about 10%, whereas the SASA of the hydrophilic and polar residues increased by 7% and 11%, respectively.

A representative conformation of the folded ORF6 for the subsequent simulations was selected based on the cluster analysis of the folding trajectory with a cut-off of 3.8 Å. The results are presented in Supplementary Figure S4. A total of 51 clusters were identified with the chosen cut-off, with the five largest clusters capturing 89% of the conformations. Notably, within the first 1.5 µs, the protein settled in a local energy minimum and explored conformations that all belonged to the largest identified cluster. Its centroid conformation is shown in Supplementary Figure S5.

### 2.2. Structure of ORF6 in an ER Membrane

ORF6 is considered a membrane protein [15,26,27,28]; therefore, the obtained structure was inserted into a lipid bilayer, modelling an ER membrane, the protein C-terminus being placed at the outer leaflet of the membrane (Supplementary Figure S6). The thus obtained conformation was subjected to a 1.4 µs production MD simulation.

Throughout the simulation, the ORF6 molecule remained largely organized as an α-helical bundle (see secondary structure plot in Supplementary Figure S7); however, the different helices underwent some changes in their mutual orientation. Experimental data show that the part of the ORF6 protein that is responsible for its biological activity is its C-terminus, so it is expected to be flexible and solvent exposed. Hence, a conformation of interest should be one in which the C-terminal tail of ORF6 is not buried in the membrane, but remains freely exposed to the solvent. Initially, the C-terminus (aa 41–61) was partly structured into an α-helix, which started to unwind and become disordered after about 1.16 µs as it began to stick up from the lipid bilayer. This is manifested in the secondary structure plot in Supplementary Figure S7. The exit of the C-terminus from the membrane is also evident from the evolution of the gyration radius of the protein (Supplementary Figure S8a): after 1.16 µs the initial value of 1.15 nm was almost doubled to nearly 2.1 nm at the end of the simulation as the C-terminal tail unfolded and started to sway around into the solvent bulk. The RMSF analysis (Supplementary Figure S8b) showed that while the N-terminal part of ORF6 (aa 1–40) remained quite stable, the C-terminus was its most flexible part both prior to and after its unfolding and sticking out into the solvent when the fluctuations of the positions of its residues doubled. After about 300 ns, additional MD simulations were performed The MD simulation was carried out for further ca. 300 ns to ensure that a stable conformational state was achieved with only the C-terminal part of ORF6 outside of the membrane and not the whole protein. As evident in the SASA plot (Supplementary Figure S9), the α-helical globular part of the protein (aa 1–40) remained firmly submerged in the membrane and not solvent exposed, while the SASA of the C-terminus doubled from about 12.5 nm${}^{2}$ to about 24.8 nm${}^{2}$ at the end of the simulation. Thus, using molecular modelling, we showed that it is entirely possible that ORF6 is an integral monotopic membrane protein and not just a membrane peripheral protein [29]. The final conformation of this simulation was used as an input model for studying the interaction of ORF6 and RAE1 (Supplementary Figure S10).

### 2.3. Interaction of RAE1 and ORF6

The RAE1 binding propensity with respect to the SARS-CoV-2 ORF6 protein was probed in a simulation with the starting conformation shown in Supplementary Figure S11. The RAE1 structure was completed and simulated for 100 ns as described in Section 4.3, the generated trajectory being subjected to a cluster analysis with a 0.25 nm cut-off (Supplementary Figure S12). The centroid of the largest cluster (Supplementary Figure S12b) was used as a model for the RAE1 protein in the interaction simulation. A 630 ns production MD simulation was carried out with this system.

**Figure 1:**
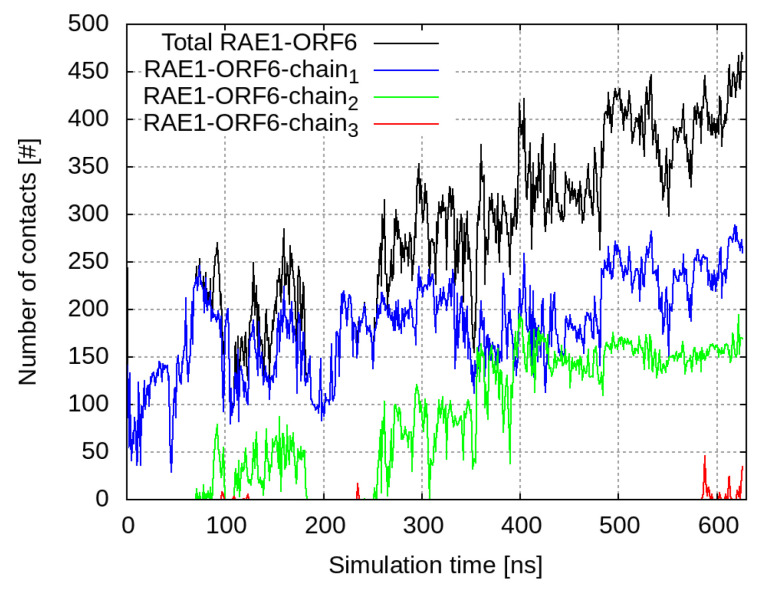
Number of contacts within 6 Å between RAE1 and the four ORF6 molecules. The total number of contacts between all ORF6 molecules and RAE1 is shown in black, and contacts with individual ORF6 chains are represented, respectively, with blue, green and red curves.

**Figure 2:**
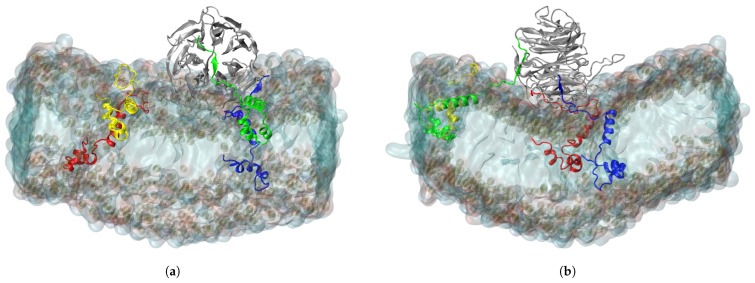
(**a**) X- and (**b**) Y-projections of RAE1, bound to three ORF6 molecules. The RAE1 protein is the gray cartoon representation and the four ORF6 molecules are the blue, green, red and yellow cartoon representations, respectively.

The C-termini of the ORF6 molecules started to interact with the nearby RAE1 almost immediately. Out of the four viral protein molecules, three form close contacts (i.e., heavy atoms from the ORF6 molecules are within a cut-off distance of 6 Å to heavy atoms of the RAE1 molecule) with the mRNA export factor (Figure 1). The final conformation in the RAE1–ORF6 simulation is presented in Figure 2.

**Figure 3:**
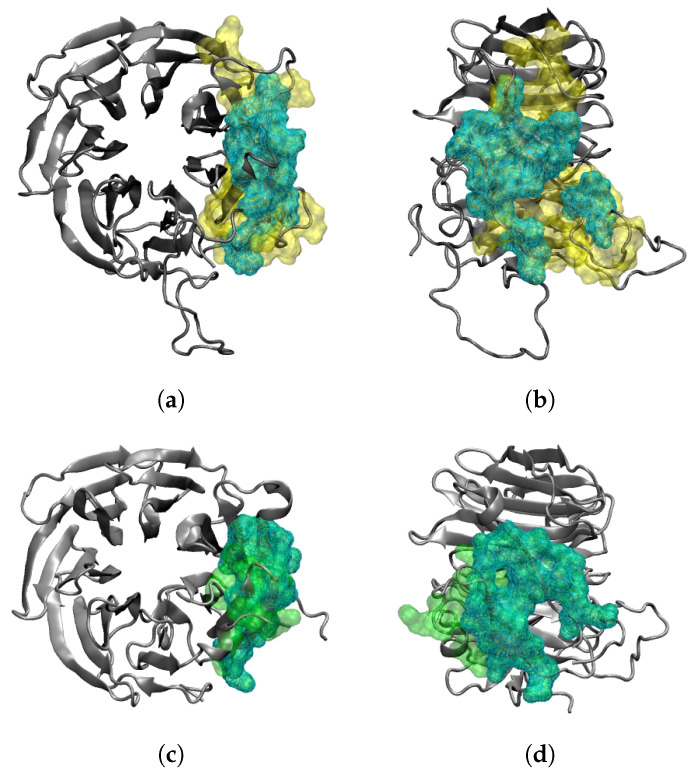
Comparison between the RAE1–ORF6 binding interfaces obtained (**a**,**b**) in the last 100 ns of the simulation (yellow surface representation) and (**c**,**d**) experimentally in PDB IDs 7VPH and 7F60 (green surface representation). The RAE1 protein is the gray cartoon, and the shared binding interface is represented by a cyan wireframe surface.

At the beginning of the simulation, the total number of RAE1–ORF6 contacts was fully determined by the binding of the first ORF6 molecule, represented in blue in Figure 1 and Figure 2. ORF6 chain${}_{1}$ bound spontaneously to RAE1, with some of the binding site residues being Ile${}^{207}$–Leu${}^{211}$, Gly${}^{239}$, Ile${}^{242}$–His${}^{243}$, Asn${}^{254}$–Lys${}^{258}$, Trp${}^{300}$, Arg${}^{305}$–Leu${}^{308}$ and Thr${}^{310}$ (Figure 3a,b). Recently, two experimental structures of the interaction between the end of the ORF6 C-terminus and the RAE1–NUP98 complex have become available [16,30]. A comparison of the experimentally and computationally obtained RAE1–ORF6 interaction interfaces is shown in Figure 3. The binding site of the first ORF6 molecule and RAE1 identified in our simulation agrees excellently with the experimentally observed one.

**Figure 4:**
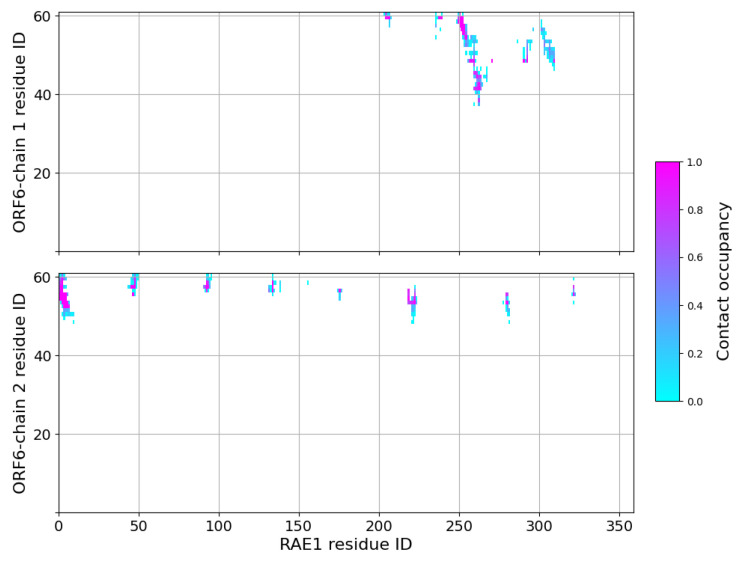
Contact maps in the last 100 ns of the RAE1–ORF6 binding simulation. A contact is considered present if two heavy atoms from the two binding partners are within a cut-off of 6 Å.

Shortly after the start of the interaction with the first protein, a second ORF6 chain bound to the RNA transport protein at residues Phe${}^{4}$–Ser${}^{13}$, Pro${}^{49}$–Thr${}^{51}$, Ser${}^{95}$–Asp${}^{97}$, His${}^{135}$–Pro${}^{139}$, Ser${}^{142}$, Ile${}^{179}$–Tyr${}^{180}$, Lys${}^{222}$–Lys${}^{227}$, Pro${}^{283}$–His${}^{285}$ and His${}^{325}$–Asn${}^{326}$. This binding surface lay at the entrance of the central tunnel of the RAE1 beta-propeller (Supplementary Figure S13). Binding occurred on the opposite side of the NUP98 recruitment site of the RAE1 molecule [16,30,31,32]. The third ORF6 chain interacted sporadically with RAE1 residues Thr${}^{22}$–Thr${}^{23}$, Thr${}^{35}$–Ser${}^{36}$ and Thr${}^{77}$–Pro${}^{79}$. The contact interfaces between ORF chains 1 and 2 and RAE1 in the last 100 ns of the simulation are shown in Figure 4.

The results of this in silico study suggest that ORF6 inserted in the membrane of the ER, as well as possibly the GA and other membrane organelles, interacts with RAE1 and binds to it to form a very stable complex. In fact, a single RAE1 molecule is able to simultaneously bind to several ORF6 molecules, which completely immobilizes the transport factor on the cytoplasmic membrane. Thus, it is reasonable to explain some of the pathogenic effects of SARS-CoV-2 ORF6 by this interaction, which anchors available cytoplasmic RAE1 proteins to the ER, GA and/or other membrane organelles, thereby depleting RAE1 availability in the nucleus and restricting mRNA transport.

### 2.4. Co-Localization between RAE1 and ORF6

**Figure 5:**
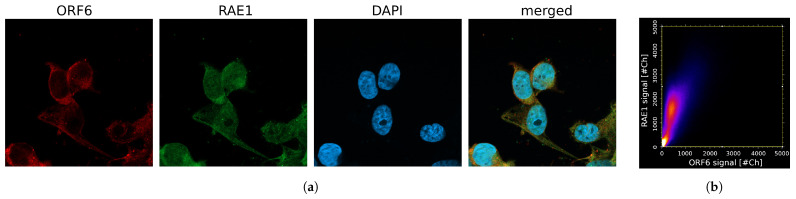
ORF6 co-localizes with RAE1. (**a**) Confocal images of cells transfected with the ORF6-expressing plasmid stained with antibodies against RAE1 and ORF6. (**b**) Co-localization scatterplot: R = 0.73, M1 = 0.99, M2 = 0.98.

To experimentally test our model, we transfected PC3 and WISH cell lines with a plasmid, constitutively expressing ORF6. Confocal microscopy (Figure 5a and Supplementary Figure S14) indicated the co-localization of ORF6 and RAE1 proteins (Figure 5b) in both cell lines, which changes RAE1 localization from mainly nuclear to predominantly cytoplasmic.

**Figure 6:**
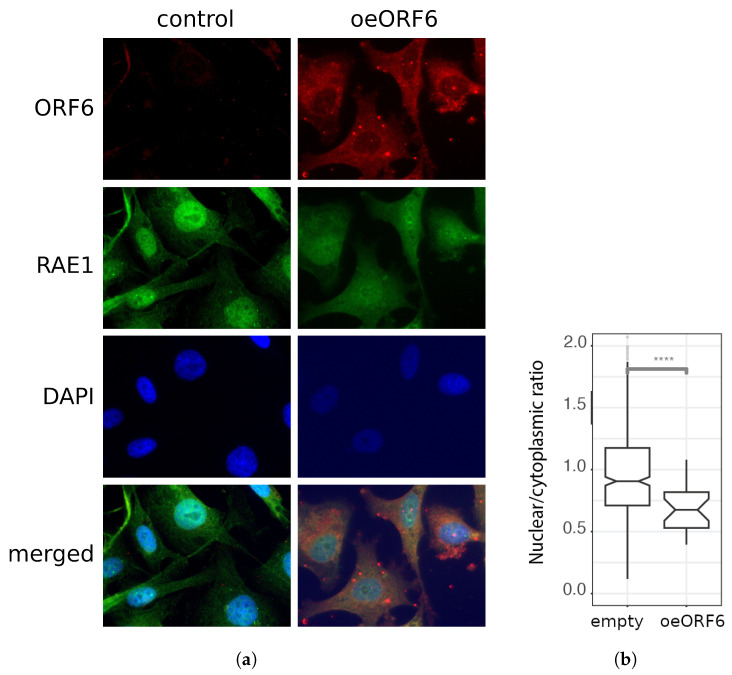
ORF6 alters the subcellular localization of RAE1. (**a**) Control (transfected with empty plasmid) and ORF6-overexpressing cells (oeORF6) were stained with antibodies against RAE1 and ORF6. Representative images are shown. (**b**) Nucleus-to-cytoplasm ratio of cells from (**a**) was calculated after measuring the fluorescence intensity of RAE1 staining using CellProfiler software. The difference is statistically significant with a **** *p*-value < 0.0001, estimated using a Student’s test for three independent experiments (two-tailed unpaired Student’s test).

An analysis of the fluorescence intensity of RAE1 (Figure 6a,b) in the nuclei and the cytoplasms of control and transfected cells showed that the nucleus-to-cytoplasm ratio of the RAE1 signal is significantly reduced in cells overexpressing ORF6. This supports the hypothesis that ORF6 alters the cellular localization of RAE1 by anchoring it in the cytoplasm.

### 2.5. Overexpression of SARS-CoV-2 ORF6 Inhibits Cell Cycle Progression

**Figure 7:**
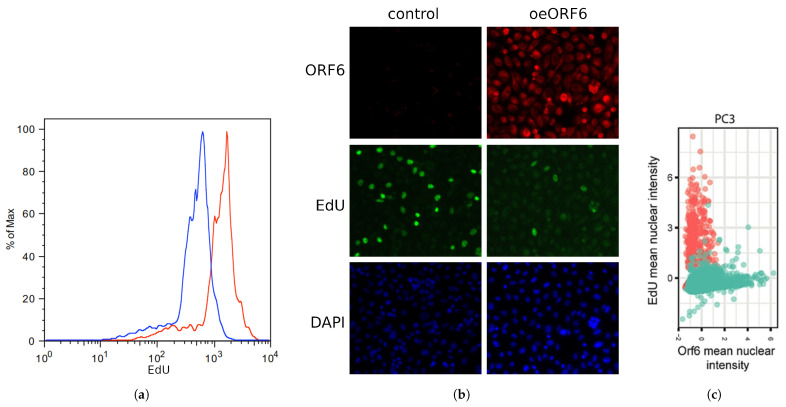

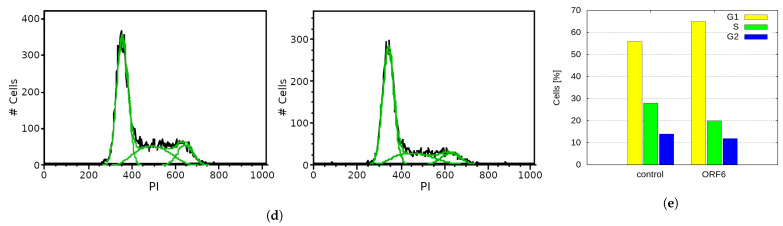
ORF6 overexpression inhibits cell cycle progression. (**a**) Control and ORF6-expressing cells were labelled with EdU and stained with AlexaFluor488-azide by a “click” reaction. Fluorescence staining of cells was assessed by flow cytometry (blue line—control cells, red line—ORF6-overexpressing cells). (**b**) Control and ORF6-expressing cells labelled with EdU were co-stained with an antibody against ORF6 and AlexaFluor488-azide. Representative images are shown. (**c**) Dot plot to compare fluorescence intensities of EdU and ORF6 for each cell. Red dots—control cells; Green dots—ORF6-overexpressing population. Similar results were obtained in three independent experiments. (**d**) Cell cycle profiles of control and ORF6-expressing cells. Black line—experimental data, green line—Dean-Jett-Fox modelling of the cell cycle phases (done in FlowJo). (**e**) Cell cycle distribution of cells from (**d**). Similar results were obtained in at least three independent experiments.

ORF6 was shown to be the most toxic SARS-CoV-2 protein [19]. However, the mechanism of its toxicity is yet unknown because the findings rely on an assay that quantifies metabolically active cells [19]. To shed light on the processes underlying ORF6’s toxicity, we studied the effect of ORF6 overexpression on DNA replication. To this end, ORF6-overexpressing and control cells were labelled with 5-Ethynyl-2’-deoxyuridine (EdU), a thymidine analogue, incorporated into DNA during replication [33]. After staining the incorporated EdU via “click” chemistry and subjecting cells to flow cytometry, we observed a leftward shift of the peak of the ORF6-expressing cells (Figure 7a). This indicates a weaker incorporation of the thymidine analogue in these cells. To further confirm that ORF6 causes replication impairment, we co-stained the EdU-labelled control and ORF6-expressing cells with AlexaFluor488-azide and an antibody against ORF6 (Figure 7b). The analysis of the mean immunofluorescence intensity of EdU and ORF6 clearly showed that the level of incorporation of EdU anticorrelates with the levels of ORF6 (Figure 7c). This suggests that ORF6 impairs DNA replication.

To further investigate the observed effect, we analyzed the cell cycle of ORF6-overexpressing cells by flow cytometry after propidium iodide (PI) staining. The cell cycle profiles of the transfected cells (Figure 7d,e) indicated a significant decrease in the percentage of cells in the S- and the G2-phases of the cell cycle. This is an indication that ORF6 impedes S-phase entry.

### 2.6. ORF6 Overexpression Inhibits Proliferation by Decreasing Cyclin E Levels

**Figure 8:**
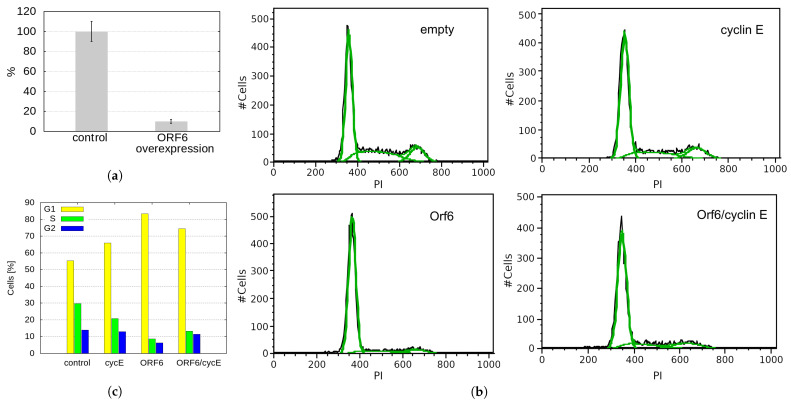
ORF6 overexpression reduces cyclin E levels and S-phase cells. (**a**) Quantitative RT-PCR analysis of the levels of cyclin E in control and ORF6-overexpressing cells (ORF6 OE). (**b**) Cell cycle profiles of control cells (transfected with empty vector) and cells transfected with plasmids expressing ORF6, cyclin E or both simultaneously. To maintain the same expression in single and double plasmid transfections, in cyclin E and ORF6 single transfections, half of the plasmid was empty. Black line—experimental data, green line—Dean-Jett-Fox modelling of the cell cycle phases (done in FlowJo). (**c**) Cell cycle distribution of cells from (**b**). Similar results were obtained in at least three independent experiments.

The progression of cells through the different stages of the cell cycle is regulated by a family of regulatory proteins called cyclins. Cyclin E is a limiting factor for G1 phase progression and S phase entry [34]. It has been shown that *Drosophila melanogaster* RAE1 is required for normal proliferation. Depletion of dmRAE1 inhibited the progression through the G1 phase of the cell cycle and cells failed to enter the S-phase due to impaired cyclin E expression [35]. As ORF6 forms a complex with RAE1, it could conceivably sequester the latter (mimicking RAE1 depletion), reduce the level of cyclin E and prevent cells from entering the S-phase. To test this hypothesis, we performed qRT-PCR to monitor the cyclin E levels in cells transfected with ORF6. As a result, we observed a ten-fold decrease in the mRNA levels of cyclin E in cells overexpressing ORF6 (Figure 8a), which could explain the depletion of cells in the S-phase.

To functionally test if cyclin E depletion in ORF6-expressing cells causes cell cycle defects, we checked if it could be rescued by cyclin E overexpression. A cell cycle analysis of cells co-transfected with cyclin E and ORF6 showed that cyclin E expression partially unblocked cell cycle progression (Figure 8b,c). This indicates that the ORF6-induced sequestration of RAE1 and the consequent cyclin E decrease significantly contribute to the defective proliferation of cells expressing ORF6.

### 2.7. ORF6 Overexpression Causes Accumulation of R-Loops, Which Impedes Progression of Active Replication Forks

**Figure 9:**
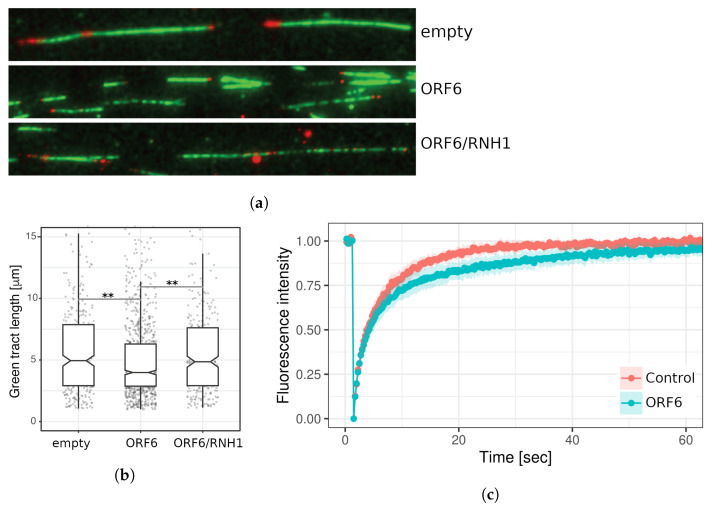
ORF6 reduces the replication fork rate as a consequence of R-loop accumulation. (**a**) DNA fibres from control (transfected with empty plasmid), ORF6-expressing plasmid and a plasmid expressing ORF6 and RNaseH1 simultaneously. (**b**) Replication rates cells from (**a**), ** *p*-value < 0.01, based on three independent experiments (two-tailed unpaired Student’s *t*-test). (**c**) FRAP analysis of DsRed–RLB in control and ORF6-overexpressing cells.

Next, we investigated the effect of ORF6 overexpression on the progression of individual replication forks. To this end, 24 h after transfection with the ORF6-expressing plasmid, cells were subjected to a DNA fibre labelling analysis measuring the lengths of second label (green) segments of red-green tracks. The data (Figure 9a,b) indicated that individual replication fork rates were reduced in cells expressing ORF6. The reduced rate of forks could be explained by impediments that stall their progression.

Defects in mRNA export lead to transcription-dependent genomic instability in yeast and metazoans [36,37,38] as a consequence of the accumulation of cotranscriptional R loops which interfere with DNA replication [39], ultimately leading to genomic instability [40,41,42].

Since the interaction of ORF6 with RAE1 disrupts the mRNA transport from the nucleus to the cytoplasm, we asked whether R-loops could be the cause of the DNA replication defect in ORF6-expressing cells. To functionally test this hypothesis, we co-transfected cells with the ORF6-expressing plasmid and a plasmid overexpressing the endonuclease RNAse H1, which specifically targets and removes RNA–DNA hybrids from the genome. An assessment of the DNA synthesis rates evaluated using DNA fibre labelling indicated that RNAseH1 rescued fork rates in replicating cells (Figure 9a,b). This suggests that R-loops are the likely cause of replication impediments in ORF6-expressing cells. To confirm that ORF6 overexpression indeed causes R-loop accumulation, we took advantage of a construct expressing the RNA binding domain of RNAse H1 fused to DsRed (RBD–DsRed). The binding of this artificial protein to R-loops allows assessment of their abundance in living cells by FRAP analysis. The result (Figure 9c) indicated that the rate of fluorescence recovery was lower in cells expressing ORF6. The reduction in the mobile fraction of RBD–DsRed observed in these cells indicated that they contain more R-loops than the controls.

## 3. Discussion

SARS-CoV-2 ORF6 is an excellent target for drug design aimed at reducing the pathogenic effects of the virus. Modern rational drug design approaches require detailed knowledge of the structure and interactions of the target protein. However, ORF6 remains one of the few SARS-CoV-2 proteins that still lack experimentally resolved 3D structures [2]. Currently, there are two entries in the protein data bank (PDB IDs 7VPH and 7F60) that provide experimental data on the interaction of the last dozen C-terminal residues of ORF6 and its target protein, the mRNA export factor RAE1 [16,30].

Existing data show that ORF6 is localized on cytoplasmic membranes (ER, GA, autophagosomes and lysosomes) [13,19,29]. Here, we present a full-length structural model of the SARS-CoV-2 ORF6 protein inserted in a model ER membrane. While the C-terminus of the accessory protein comes out of the lipid bilayer and moves around fairly freely in the solvent, the N-terminal part of the molecule remains quite stably integrated into the membrane. Our results strongly support the hypothesis that ORF6 is an integral monotopic membrane protein rather than a peripheral membrane protein [29].

Numerous studies have demonstrated that the protein interferes with the RAE1–NUP98 complex, a component of the nuclear pore complex [15,16,27,28], altering the localization of RAE1, which consequently impairs mRNA transport [28,43]. Hall et al. [44] showed that ORF6 does not interact directly with NUP98 and that mRNA export blockage is dependent only on the interaction with RAE1. It is noteworthy that other viruses, such as the vesicular stomatitis virus (VSV) and the herpesvirus, employ the same strategy to disrupt nuclear mRNA export by targeting RAE1 [32,45].

Here, the formation of a stable ORF6–RAE1 protein complex was supported directly by in silico experiments and indirectly by in vitro data from transfected PC3 and WISH cell lines. Notably, this process caused RAE1 to localize predominantly in the cytoplasm instead of in the nucleus.

A model system containing four ORF6 molecules inserted in an ER membrane, with one RAE1 protein placed in close proximity to them, was used to elucidate the molecular basis of the SARS-CoV-2 ORF6–RAE1 binding and the changes in RAE1 subcellular localization by means of MD simulations. The simulations revealed that RAE1 immediately engages with at least one ORF6 protein. The contact surface of this interaction coincides to a great extent with the binding interface of RAE1 to the M-protein of the VSV [32] (see Supplementary Figure S15). A similar binding mode had been observed for the herpesvirus ORF10 protein and mouse RAE1 [45]. This surface groove on the side of its beta-propeller was found to actually be the RNA binding site of the RAE1 molecule [31,32]. Importantly, Met${}^{58}$ in the C-terminus of ORF6-chain${}_{1}$ interacts with residues Phe${}^{255}$, Phe${}^{257}$, Trp${}^{300}$ and Arg${}^{305}$ that lie in a deep side pocket of the RAE1 beta-propeller in a manner similar to VSV M-protein. The M-cavity formed by these residues strictly selects a methionine residue and favors binding of sequences containing a methionine flanked by acidic residues that form salt bridges with positively charged residues (lysine and arginine) in the RNA-binding groove of RAE1 [30]. The same contact interface (239–240, 242, 254–259, 261, 271, 300, 305–312) was experimentally found to be the binding site for both the SARS-CoV ORF6 and SARS-CoV-2 ORF6 C-terminal peptides [16,30]. Thus, our modelling results are in excellent agreement with the available experimental data.

In addition to the first ORF6, in our simulation system, RAE1 also interacts with two more ORF6 molecules. The second molecule binds the transport protein at the entrance of the central tunnel of the RAE1 beta-propeller that is opposite to the NUP98 binding site. Occasionally, RAE1 also forms some transient contacts with a third ORF6 C-terminus, involving residues in its N-terminus that is positioned in close proximity to the model membrane.

Our in vitro data fully support the observations from the MD simulations about the co-localization of ORF6 and RAE1, which leads to a significant depletion in RAE1 in the nuclei of cells transfected with ORF6. Thus, our results suggest a model in which ORF6 interacts with RAE1 in the cytoplasm, preventing RAE1 from binding to mRNA and possibly to NUP98 in the nuclear pores.

It is very probable that the toxicity of ORF6—the highest of all SARS-CoV-2 proteins with a reduction in cell viability of over 50% [19]—is rooted in the observed sequestering of RAE1 into the cytoplasmic membranes. RAE1 sequestration also underlies the effects we observed on cell cycle progression and replication fork speeds, contributing to ORF6’s cytotoxicity. ORF6 overexpression impairs DNA replication, as evidenced by the weaker incorporation of the thymidine analogue EdU and the correlation between EdU incorporation and ORF6 levels. A cell cycle analysis reveals that ORF6 hinders cell cycle progression, with a significant decrease in the percentage of cells in the S- and G2-phases, suggesting that ORF6 interferes with S-phase entry.

A possible explanation for ORF6-mediated cell cycle defects lies in its effect on cyclin E levels. As cyclin E is a key regulator of G1-phase progression and S-phase entry, the observed ten-fold decrease in mRNA levels of cyclin E in ORF6-overexpressing cells could be responsible for the observed depletion of cells in the S-phase. The partial rescue of cell cycle progression upon co-transfection of cyclin E and ORF6 supports the hypothesis that ORF6-induced sequestration of RAE1 and the resulting decrease in cyclin E underlie the defective proliferation of cells expressing ORF6. These data are in line with the report by Sitterlin [35] that the presence of dmRAE1 is required for normal proliferation and, more importantly, for normal cyclin E expression, suggesting that the human homologue, hsRAE1, may also play a similar role in the cell cycle.

Impaired nuclear export has been linked to accumulation of R-loops [46,47,48], which could stall DNA replication forks [49,50,51]. The obstruction of RNA export is likely the cause of R-loop accumulation, known to cause replication stress, which we observe in FRAP experiments as a lower mobile fraction of RBD–DsRed in ORF6-expressing cells. A link between R-loops accumulating in ORF6-expressing cells and impeded replication is provided by the observation that RNAseH1 overexpression rescued replication fork rates. A similar effect has been described in which Kaposi’s sarcoma herpesvirus sequesters the transcription and export factor TREX, leading to R-loops and genome instability [52].

Thus, our study provides evidence for two ways in which SARS-CoV-2 ORF6 protein could affect cellular proliferation: by inhibiting cell cycle progression and by accumulation of R-loops. A very recent paper has demonstrated that SARS-CoV-2 proteins ORF6 and Nsp13 cause degradation of the DNA damage response kinase CHK1 through proteasome and autophagy, respectively. CHK1 loss leads to a deoxynucleoside triphosphate shortage, causing impaired S-phase progression, DNA damage, pro-inflammatory pathways activation and cellular senescence [53]. Using a DNA fibre labelling analysis, we observed that the supplementation of deoxynucleoside triphosphates rescued the progression of replication forks in cells overexpressing ORF6 (data not shown), thus confirming the research mentioned above. This and the results reported here indicate the broad range of mechanisms by which SARS-CoV-2 influences cell proliferation and genome integrity.

## 4. Materials and Methods

### 4.1. MD Simulation Protocol

All MD simulations were performed using the GROMACS simulation package [54], version 2019.6 and later. The CHARMM36 force field was used for parameterization of the proteins and lipids in the membrane [55,56]. All simulations were performed in explicit water solvent with a concentration of NaCl of 0.15 mol/L. The energy of the systems was minimized using the steepest descent algorithm with a maximum force tolerance of 100 kJ/(mol·nm). Then, a short 50 ps position-restraint simulation was performed to equilibrate the solvent, followed by a 10 ns isothermal-isobaric simulation, equilibrating the temperature at 310 K and the pressure at 1 atm using the Berendsen thermostat and barostat [57]. For production simulations in the NTP ensemble, the temperature was maintained constant at 310 K using the v-rescale thermostat [58] with a coupling constant of 0.2 ps, and the pressure was maintained constant at 1 atm using the Parrinello–Rahman barostat [59] with a coupling constant of 2 ps. The leap-frog integrator was used with a timestep of 2 fs, allowed for by constraining the bonds between heavy atoms and hydrogens with the PLINCS algorithm [60]. The PME method with a direct cut-off of 1.2 nm was used for calculations of the electrostatic interactions. van der Waals forces were smoothly switched off from a distance of 1.0 nm and truncated at 1.2 nm. Neighbourlists were updated every 10 timesteps.

### 4.2. Input Structural Models

#### 4.2.1. SARS-CoV-2 ORF6 Protein

The 3D structure of the SARS-CoV-2 ORF6 has not yet been resolved. As an input for our simulations we used a 3D model of the protein, developed by the EXCALATE4COV project and available in their open access repository [61]. This model was developed using 10 µs long MD folding simulations, starting from a molten globule model conformation that consisted of three α-helical segments. We further performed additional 7.6 µs of folding simulation of this structure using the simulation protocol described in Section 4.1. The protein was solvated in a cubic box with a 1.2 nm minimal distance to the box walls. Trajectory frames were written every nanosecond.

#### 4.2.2. SARS-CoV-2 ORF6 Embedded in a Model ER Membrane

As an input structure for the development of this model, the centroid of the largest cluster of the trajectory from Section 4.2 (with a cut-off of 3.8 Å) was inserted in a lipid bilayer with the membrane composed of 54% POPC, 20% POPE, 11% POPI and 8% cholesterol, to model the content of endoplasmic reticulum membranes, according to [62] and references therein. For generation of the lipid positions and ORF6 insertion in the bilayer, the Membrane/Bilayer Builder module [63] of the CHARMM-GUI server [64] was used. The lipid bilayer had an area of 76.3 × 76.3 Å${}^{2}$ and contained 192 molecules in total. A 1.4 µs long production MD run was performed, with trajectory frames put out every nanosecond.

### 4.3. Modelling the Interaction of SARS-CoV-2 ORF6 and RAE1

The obtained stable structure of the ORF6 protein in a model ER membrane was duplicated in both directions in the plane of the lipid bilayer, which resulted in a membrane of size 150 × 150 Å${}^{2}$ containing a total of 769 lipids and four ORF6 molecules inserted into them. As an input structure for the RAE1 protein, we used chain A of the crystallographic structure with PDB ID 3MMY [31]. The missing loop residues (aa 19–22 and 264–267) were reconstructed using the macromolecular crystallography and model-building toolkit COOT [65]. This RAE1 initial model was simulated for 100 ns. The centroid of the largest cluster of the equilibration trajectory was placed at a distance of about 7 Å above the membrane between the fluttering C-termini of the four ORF6 molecules. A 630 ns production MD simulation was carried out in order to study the interaction of the RNA carrier protein and the viral accessory proteins.

### 4.4. MD Data Analysis

The MD trajectories were postprocessed and analyzed using the standard GROMACS postprocessing and analysis tools for calculations of RMSD, RMSF, SASA, Rg and cluster analysis. Least-square fitting to the initial conformation was performed prior to all analyses in order to remove all global translational and rotational movements. The gromos algorithm [66] was used for cluster analyses. The secondary structure of the proteins was assigned by the STRIDE algorithm [67] as implemented in the visualization and manipulation package VMD [68]. All structural figures were also generated with VMD.

### 4.5. Cell Culture, Plasmids and Antibodies

WISH (ATCC®CCL-25^™^, ATCC, Manassas, VA, USA) and PC3 (ATCC®CRL-1435^™^) cell lines were propagated in MEM and DMEM, respectively, supplemented with 10% fetal bovine serum (Gibco^™^, Waltham, MA, USA) and penicillin-streptomycin (10,000 U/mL, Gibco^™^, Waltham, MA, USA). Cells were cultured in a humidified incubator at 37 °C and 5% CO_2_.

For the overexpression of SARS-CoV-2 ORF6 protein, the plasmid pLVX-EF1alpha-SARS-CoV-2-orf6-2xStrep-IRES-Puro (Addgene plasmid # 141387 from Nevan Krogan) [26] was used. The cyclin E-expressing vector was a gift from Bob Weinberg (Addgene # 8963).

The RBD–DsRed plasmid was constructed by PCR cloning the HB domain of RNAse H1 into the pDsRed-Express-C1 vector (Clontech, Mountain View, CA, USA) as earlier described [69].

### 4.6. Quantitative Real-Time PCR Analysis

Total RNA from cells overexpressing ORF6 was extracted using TRIzol reagent (Invitrogen, Carlsbad, CA, USA). The concentration and purity of extracted RNA were determined by a Nanodrop-1000 (Thermo Fisher, Waltham, MA, USA). RNA integrity and quality were assessed using 1% agarose gel electrophoresis in TAE buffer (40 mM Tris-acetate, 1 mM EDTA). Subsequently, 1 µg total RNA from each sample was subjected to cDNA synthesis via a RevertAid H Minus First Strand cDNA Synthesis Kit (Thermo Scientific^™^, Waltham, MA, USA) according to the manufacturer’s recommendations. The relative expression levels of target genes (RAE1 and Cyclin E) were assessed by a qRT-PCR analysis using the SYBR^™^ Select Master Mix (Thermo Scientific^™^). Housekeeping gene β-actin was used as an endogenous control to normalize gene expressions.

Primer oligonucleotide sequences of the studied genes are listed in Supplementary Table S1. The analysis was performed on a Rotor-Gene 6000 thermal cycler (Corbett, QIAGEN, Hilden, Germany). Gene expression data were analyzed using Rotor-Gene 6000 Software (QIAGEN) and the relative expression levels of the genes of interest were normalized to the endogenous control for each sample. Each qRT-PCR reaction was performed in at least three replicates in different PCR runs. Statistical differences were evaluated using a *t*-test and values at <0.05 were considered as statistically significant.

### 4.7. 5-Ethynyl-2’-deoxyuridine (EdU) Labelling

Cells were incubated with 25 µM EdU for 20 min immediately before fixation. A “click” reaction was carried out using a Click-iT^™^ EdU AlexaFluor^™^ 488 kit according to the manufacturer’s protocol (Thermo Scientific^™^). When combined with immunostaining, the “click” reaction was performed immediately after secondary antibody incubation.

### 4.8. Flow Cytometry

To analyze cell cycle profiles, approximately 5 $\times \;10^{5}$ cells were trypsinized, harvested by centrifugation for 10 min at 400 g and fixed in 70% ethanol. Before analysis, cells were resuspended in 1 × PBS, treated with RNAse A (20 µg/mL) and stained with propidium iodide (20 µg/mL). Analyses were carried out with FACScalibur apparatus with Cellquest (Becton Dickinson, Franklin Lakes, NJ, USA) and FlowJo software.

### 4.9. Immunofluorescent Microscopy

WISH and PC3 cells were cultured on *⌀* 12 mm coverslips (Epredia, Kalamazoo, MI, USA) and transfected with plasmids, relevant to the experiment being performed. Cells were fixed with either 3.7% formaldehyde in 1 × PBS for 10 min at room temperature or with methanol for 10 min at −20 °C. Fixed cells were permeabilized with 0.5% Triton X-100 in PBS for 5 min and then blocked in blocking buffer (5% BSA and 0.1% Tween 20 in 1 × PBS) for 1 h. Cells were then incubated overnight at 4 °C with primary antibodies (in blocking buffer). After washing, cells were stained with a secondary antibody for 1 h at room temperature.

The nuclei were counterstained with DAPI (Cell Signaling Technology, Danvers, MA, USA) to a final concentration of 0.5 µg/mL for 2 min at room temperature. The coverslips were washed and mounted using ProLong^™^ Gold Antifade mounting media (Invitrogen). The cells were imaged with a Zeiss Axiovert 200M fluorescence inverted microscope and images were analyzed by CellProfiler software [70].

### 4.10. Fluorescence Recovery after Photobleaching (FRAP) Analysis

Cells were transfected with the RBD–DsRed plasmid expressing the RNA binding domain of RNAse H1 fused to DsRed. An FRAP analysis was carried out using an Andor Revolution XDI spinning disk confocal system with a heated chamber in CO_2_-independent medium. Imaging was carried out in 1 s intervals for 150 s with the bleaching pulse applied at the fifth second. Intensity measurements and analyses were carried out with CellTool software [71].

### 4.11. DNA Fibre Labeling

DNA fibre analyses were performed following the standard protocol [72] with slight modifications. Briefly, exponentially growing PC3 cells were first incubated with 25 µM chlorodeoxyuridine (CldU) for 10 min and then with 250 µM iododeoxyuridine (IdU) for 25 min both at 37 °C and 5% CO_2_. Spreads were prepared from 2500 cells and suspended in 1 × PBS at $1\times 10^{6}$ cells/ml. Cell lysis was carried out in fibre lysis solution (50 mM EDTA and 0.5% SDS in 200 mM Tris-HCl, pH 7.5). DNA fibres were spread by tilting the slides ∼25° until the drop of the fibre solution reached the bottom of the slide and then letting it dry. Dried slides were immersed in 2.5 M HCl for 80 min, washed in PBS and blocked for 40 min in 5% BSA in 1 × PBS. Primary antibodies—mouse anti-BrdU antibody (Becton Dickinson, cat # 347580) to detect IdU and rat anti-BrdU antibody (Abcam, Cambridge, UK cat # Ab6326) to detect CldU—were diluted in blocking buffer and applied overnight. After washing, slides were incubated with secondary antibodies goat anti-rat DyLight®594 (Abcam 96889) and goat anti-mouse DyLight®488 (Abcam, 96879) for 60 min. Slides were mounted with ProLong Gold anti-fade reagent (Invitrogene). Images were acquired with an Axiovert 200 M microscope (Carl Zeiss, Jena, Germany) equipped with an Axiocam MR3 camera (Carl Zeiss). Fibre length measurements were carried out using DNA size finder software, version 1.0 [73].

## 5. Conclusions

In this report, we studied the interaction of SARS-CoV-2 ORF6 and RAE1 proteins. Our in silico data demonstrate that RAE1 is able to bind simultaneously to multiple C-termini of ORF6 molecules. These interactions anchor the transport protein to cytoplasmic membranes, thus sequestering it in the cytoplasm of the host cell and depleting its availability in the nucleus. All this results in disrupting of nucleocytoplasmic trafficking. These results were confirmed experimentally by the observed ORF6–RAE1 colocalization and the change in RAE1 localization from mainly nuclear to predominantly cytoplasmic. Our results suggest for the first time a mechanism by which the interaction of ORF6 with RAE1 leads to genome instability. Firstly, this complex formation interferes with the cell cycle progression. Secondly, R-loops accumulate due to deficient mRNA transportation, leading to stalled DNA replication forks and eventually causing replication stress. Both of these effects are partially reversible by overexpression of either cyclin E, which helps the progression of the cell from the G1- to S-phase, or RNAse H1, which has the ability to remove RNA from the RNA–DNA hybrids.

This action of SARS-CoV-2 ORF6 aims at hindering the synthesis of antiviral molecules and slowing down the immune response of the host cells, but does not significantly affect viral replication, since coronaviruses do not rely on nuclear export for their replication and transcribe their genome in the cytoplasm. Therefore, targeting the interaction of the SARS-CoV-2 ORF6 C-terminus and RAE1 can potentially lower the pathogenic effects of the virus and also facilitate an earlier antiviral response, thus reducing viral replication in infected host cells. Understanding the multiple ways in which ORF6 affects DNA replication might also have important implications in clarifying the pathogenesis of SARS-CoV-2 and for the development of therapeutic strategies to counteract its deleterious effects on host cells.

## Data Availability

The data will be made available upon request.

## Supplementary Materials

The supporting information can be downloaded at: https://www.mdpi.com/article/10.3390/ijms241411589/s1.

## Abbreviations

The following abbreviations are used in this manuscript:

aa	amino acids
CHOL	cholesterol
ER	endoplasmic reticulum
GA	Golgi apparatus
MD	molecular dynamics
NUP98	nucleoporin 98
oeORF6	ORF6-overexpressing cells
ORF6	open reading frame 6
PI	propidium iodide
POPC	1-palmitoyl-2-oleoyl-sn-glycero-3-phosphocholine
POPE	1-palmitoyl-2-oleoyl-sn-glycero-3-phosphoethanolamine
POPI	1-palmitoyl-2-oleoyl-sn-glycero-3-phosphoinositol
RAE1	ribonucleic acid export 1
R${}_{g}$	radius of gyration
RMSD	root mean square deviation
RMSF	root mean square fluctuations
SASA	solvent accessible surface area

## References

[1] Wu F., Zhao S., Yu B., Chen Y.M., Wang W., Song Z.G., Hu Y., Tao Z.W., Tian J.H., Pei Y.Y. (2020). A new coronavirus associated with human respiratory disease in China. Nature.

[2] Bai C., Zhong Q., Gao G. (2022). Overview of SARS-CoV-2 genome-encoded proteins. Sci. China Life Sci..

[3] Zandi M., Shafaati M., Kalantar-Neyestanaki D., Pourghadamyari H., Fani M., Soltani S., Kaleji H., Abbasi S. (2022). The role of SARS-CoV-2 accessory proteins in immune evasion. Biomed. Pharmacother..

[4] Redondo N., Zaldívar-López S., Garrido J.J., Montoya M. (2021). SARS-CoV-2 Accessory Proteins in Viral Pathogenesis: Knowns and Unknowns. Front. Immunol..

[5] Kimura I., Konno Y., Uriu K., Hopfensperger K., Sauter D., Nakagawa S., Sato K. (2021). Sarbecovirus ORF6 proteins hamper induction of interferon signaling. Cell Rep..

[6] Grellet E., L’Hôte I., Goulet A., Imbert I. (2022). Replication of the coronavirus genome: A paradox among positive-strand RNA viruses. J. Biol. Chem..

[7] Mironov A.A., Savin M.A., Beznoussenko G.V. (2023). COVID-19 Biogenesis and Intracellular Transport. Int. J. Mol. Sci..

[8] Rashid F., Xie Z., Suleman M., Shah A., Khan S., Luo S. (2022). Roles and functions of SARS-CoV-2 proteins in host immune evasion. Front. Immunol..

[9] Zhang D., Zhu L., Wang Y., Li P., Gao Y. (2022). Translational Control of COVID-19 and Its Therapeutic Implication. Front. Immunol..

[10] Yan W., Zheng Y., Zeng X., He B., Cheng W. (2022). Structural biology of SARS-CoV-2: Open the door for novel therapies. Signal Transduct. Target. Ther..

[11] Harrison A.G., Lin T., Wang P. (2020). Mechanisms of SARS-CoV-2 Transmission and Pathogenesis. Trends Immunol..

[12] Brant A.C., Tian W., Majerciak V., Yang W., Zheng Z.M. (2021). SARS-CoV-2: From its discovery to genome structure, transcription, and replication. Cell Biosci..

[13] Frieman M., Yount B., Heise M., Kopecky-Bromberg S.A., Palese P., Baric R.S. (2007). Severe Acute Respiratory Syndrome Coronavirus ORF6 Antagonizes STAT1 Function by Sequestering Nuclear Import Factors on the Rough Endoplasmic Reticulum/Golgi Membrane. J. Virol..

[14] Kopecky-Bromberg S.A., Martínez-Sobrido L., Frieman M., Baric R.A., Palese P. (2007). Severe Acute Respiratory Syndrome Coronavirus Open Reading Frame (ORF) 3b, ORF 6, and Nucleocapsid Proteins Function as Interferon Antagonists. J. Virol..

[15] Miorin L., Kehrer T., Sanchez-Aparicio M.T., Zhang K., Cohen P., Patel R.S., Cupic A., Makio T., Mei M., Moreno E. (2020). SARS-CoV-2 Orf6 hijacks Nup98 to block STAT nuclear import and antagonize interferon signaling. Proc. Natl. Acad. Sci. USA.

[16] Li T., Wen Y., Guo H., Yang T., Yang H., Ji X. (2022). Molecular Mechanism of SARS-CoVs Orf6 Targeting the Rae1–Nup98 Complex to Compete With mRNA Nuclear Export. Front. Mol. Biosci..

[17] Yuen C.K., Lam J.Y., Wong W.M., Mak L.F., Wang X., Chu H., Cai J.P., Jin D.Y., To K.K.W., Chan J.F.W. (2020). SARS-CoV-2 nsp13, nsp14, nsp15 and orf6 function as potent interferon antagonists. Emerg. Microbes Infect..

[18] Miyamoto Y., Itoh Y., Suzuki T., Tanaka T., Sakai Y., Koido M., Hata C., Wang C.X., Otani M., Moriishi K. (2022). SARS-CoV-2 ORF6 disrupts nucleocytoplasmic trafficking to advance viral replication. Commun. Biol..

[19] Lee J.G., Huang W., Lee H., van de Leemput J., Kane M.A., Han Z. (2021). Characterization of SARS-CoV-2 proteins reveals Orf6 pathogenicity, subcellular localization, host interactions and attenuation by Selinexor. Cell Biosci..

[20] Lei X., Dong X., Ma R., Wang W., Xiao X., Tian Z., Wang C., Wang Y., Li L., Ren L. (2020). Activation and evasion of type I interferon responses by SARS-CoV-2. Nat. Commun..

[21] Zhang J., Cruz-cosme R., Zhuang M.W., Liu D., Liu Y., Teng S., Wang P.H., Tang Q. (2020). A systemic and molecular study of subcellular localization of SARS-CoV-2 proteins. Signal Transduct. Target. Ther..

[22] Morais da Silva M., Lira de Lucena A.S., Paiva Júnior S.d.S.L., Florêncio De Carvalho V.M., Santana de Oliveira P.S., da Rosa M.M., Barreto de Melo Rego M.J., Pitta M.G.d.R., Pereira M.C. (2022). Cell death mechanisms involved in cell injury caused by SARS-CoV-2. Rev. Med. Virol..

[23] Kaur M., Sharma A., Kumar S., Singh G., Barnwal R.P. (2021). SARS-CoV-2: Insights into its structural intricacies and functional aspects for drug and vaccine development. Int. J. Biol. Macromol..

[24] Netland J., Ferraro D., Pewe L., Olivares H., Gallagher T., Perlman S. (2007). Enhancement of Murine Coronavirus Replication by Severe Acute Respiratory Syndrome Coronavirus Protein 6 Requires the N-Terminal Hydrophobic Region but Not C-Terminal Sorting Motifs. J. Virol..

[25] Zhao J., Falcón A., Zhou H., Netland J., Enjuanes L., Breña P.P., Perlman S. (2009). Severe Acute Respiratory Syndrome Coronavirus Protein 6 Is Required for Optimal Replication. J. Virol..

[26] Gordon D.E., Jang G.M., Bouhaddou M., Xu J., Obernier K., White K.M., O’Meara M.J., Rezelj V.V., Guo J.Z., Swaney D.L. (2020). A SARS-CoV-2 protein interaction map reveals targets for drug repurposing. Nature.

[27] Kato K., Ikliptikawati D.K., Kobayashi A., Kondo H., Lim K., Hazawa M., Wong R.W. (2021). Overexpression of SARS-CoV-2 protein ORF6 dislocates RAE1 and NUP98 from the nuclear pore complex. Biochem. Biophys. Res. Commun..

[28] Addetia A., Lieberman N.A.P., Phung Q., Hsiang T.Y., Xie H., Roychoudhury P., Shrestha L., Loprieno M.A., Huang M.L., Gale M. (2021). SARS-CoV-2 ORF6 Disrupts Bidirectional Nucleocytoplasmic Transport through Interactions with Rae1 and Nup98. mBio.

[29] Wong H.T., Cheung V., Salamango D.J. (2022). Decoupling SARS-CoV-2 ORF6 localization and interferon antagonism. J. Cell Sci..

[30] Gao X., Tian H., Zhu K., Li Q., Hao W., Wang L., Qin B., Deng H., Cui S. (2022). Structural basis for Sarbecovirus ORF6 mediated blockage of nucleocytoplasmic transport. Nat. Commun..

[31] Ren Y., Seo H.S., Blobel G., Hoelz A. (2010). Structural and functional analysis of the interaction between the nucleoporin Nup98 and the mRNA export factor Rae1. Proc. Natl. Acad. Sci. USA.

[32] Quan B., Seo H.S., Blobel G., Ren Y. (2014). Vesiculoviral matrix (M) protein occupies nucleic acid binding site at nucleoporin pair (Rae1•Nup98). Proc. Natl. Acad. Sci. USA.

[33] Salic A., Mitchison T.J. (2008). A chemical method for fast and sensitive detection of DNA synthesis in vivo. Proc. Natl. Acad. Sci. USA.

[34] Sunwoo H.H., Suresh M.R., Wild D. (2013). Chapter 9.13—Cancer Markers. The Immunoassay Handbook.

[35] Sitterlin D. (2004). Characterization of the Drosophila Rae1 protein as a G1 phase regulator of the cell cycle. Gene.

[36] Jimeno S., Rondón A.G., Luna R., Aguilera A. (2002). The yeast THO complex and mRNA export factors link RNA metabolism with transcription and genome instability. EMBO J..

[37] Zenklusen D., Vinciguerra P., Wyss J.C., Stutz F. (2002). Stable mRNP Formation and Export Require Cotranscriptional Recruitment of the mRNA Export Factors Yra1p and Sub2p by Hpr1p. Mol. Cell. Biol..

[38] Domínguez-Sánchez M.S., Barroso S., Gímez-González B., Luna R., Aguilera A. (2011). Genome Instability and Transcription Elongation Impairment in Human Cells Depleted of THO/TREX. PLoS Genet..

[39] Gómez-González B., García-Rubio M., Bermejo R., Gaillard H., Shirahige K., Marín A., Foiani M., Aguilera A. (2011). Genome-wide function of THO/TREX in active genes prevents R-loop-dependent replication obstacles. EMBO J..

[40] Sollier J., Cimprich K.A. (2015). Breaking bad: R-loops and genome integrity. Trends Cell Biol..

[41] Gaillard H., Aguilera A. (2016). Transcription as a Threat to Genome Integrity. Annu. Rev. Biochem..

[42] Tuduri S., Crabbé L., Conti C., Tourrière H., Holtgreve-Grez H., Jauch A., Pantesco V., De Vos J., Thomas A., Theillet C. (2009). Topoisomerase I suppresses genomic instability by preventing interference between replication and transcription. Nat. Cell Biol..

[43] Pritchard C.E., Fornerod M., Kasper L.H., van Deursen J.M. (1999). RAE1 Is a Shuttling mRNA Export Factor That Binds to a GLEBS-like NUP98 Motif at the Nuclear Pore Complex through Multiple Domains. J. Cell Biol..

[44] Hall R., Guedán A., Yap M.W., Young G.R., Harvey R., Stoye J.P., Bishop K.N. (2022). SARS-CoV-2 ORF6 disrupts innate immune signalling by inhibiting cellular mRNA export. PLoS Pathog..

[45] Feng H., Tian H., Wang Y., Zhang Q., Lin N., Liu S., Yu Y., Deng H., Gao P. (2020). Molecular mechanism underlying selective inhibition of mRNA nuclear export by herpesvirus protein ORF10. Proc. Natl. Acad. Sci. USA.

[46] Skourti-Stathaki K., Proudfoot N., Gromak N. (2011). Human Senataxin Resolves RNA/DNA Hybrids Formed at Transcriptional Pause Sites to Promote Xrn2-Dependent Termination. Mol. Cell.

[47] Crossley M.P., Bocek M., Cimprich K.A. (2019). R-Loops as Cellular Regulators and Genomic Threats. Mol. Cell.

[48] Santos-Pereira J.M., Aguilera A. (2015). R loops: New modulators of genome dynamics and function. Nat. Rev. Genet..

[49] Gan W., Guan Z., Liu J., Gui T., Shen K., Manley J.L., Li X. (2011). R-loop-mediated genomic instability is caused by impairment of replication fork progression. Genes Dev..

[50] Helmrich A., Ballarino M., Tora L. (2011). Collisions between Replication and Transcription Complexes Cause Common Fragile Site Instability at the Longest Human Genes. Mol. Cell.

[51] Hamperl S., Cimprich K.A. (2014). The contribution of co-transcriptional RNA:DNA hybrid structures to DNA damage and genome instability. DNA Repair.

[52] Jackson B.R., Noerenberg M., Whitehouse A. (2014). A Novel Mechanism Inducing Genome Instability in Kaposi’s Sarcoma-Associated Herpesvirus Infected Cells. PLoS Pathog..

[53] Gioia U., Tavella S., Martínez-Orellana P., Cicio G., Colliva A., Ceccon M., Cabrini M., Henriques A.C., Fumagalli V., Paldino A. (2023). SARS-CoV-2 infection induces DNA damage, through CHK1 degradation and impaired 53BP1 recruitment, and cellular senescence. Nat. Cell Biol..

[54] Abraham M.J., Murtola T., Schulz R., Páll S., Smith J.C., Hess B., Lindahl E. (2015). GROMACS: High performance molecular simulations through multi-level parallelism from laptops to supercomputers. SoftwareX.

[55] Huang J., Rauscher S., Nawrocki G., Ran T., Feig M., de Groot B.L., Grubmüller H., MacKerell A.D. (2016). CHARMM36m: An improved force field for folded and intrinsically disordered proteins. Nat. Methods.

[56] Klauda J.B., Venable R.M., Freites J.A., O’Connor J.W., Tobias D.J., Mondragon-Ramirez C., Vorobyov I., MacKerell A.D.J., Pastor R.W. (2010). Update of the CHARMM All-Atom Additive Force Field for Lipids: Validation on Six Lipid Types. J. Phys. Chem. B.

[57] Berendsen H.J.C., Postma J.P.M., van Gunsteren W.F., DiNola A., Haak J.R. (1984). Molecular dynamics with coupling to an external bath. J. Chem. Phys..

[58] Bussi G., Donadio D., Parrinello M. (2007). Canonical sampling through velocity rescaling. J. Chem. Phys..

[59] Parrinello M., Rahman A. (1980). Crystal Structure and Pair Potentials: A Molecular-Dynamics Study. Phys. Rev. Lett..

[60] Hess B. (2008). P-LINCS: A Parallel Linear Constraint Solver for Molecular Simulation. J. Chem. Theory Comput..

[61] EXCALATE4COV Public Data Repository. https://ds-814.cr.cnaf.infn.it:8443/ex4cov-public/Molecular_Dynamics/ORF6/.

[62] Casares D., Escribá P.V., Rosselló C.A. (2019). Membrane Lipid Composition: Effect on Membrane and Organelle Structure, Function and Compartmentalization and Therapeutic Avenues. Int. J. Mol. Sci..

[63] Wu E.L., Cheng X., Jo S., Rui H., Song K.C., Dávila-Contreras E.M., Qi Y., Lee J., Monje-Galvan V., Venable R.M. (2014). CHARMM-GUI Membrane Builder toward realistic biological membrane simulations. J. Comput. Chem..

[64] Jo S., Kim T., Iyer V.G., Im W. (2008). CHARMM-GUI: A web-based graphical user interface for CHARMM. J. Comput. Chem..

[65] Emsley P., Cowtan K. (2004). Coot: Model-building tools for molecular graphics. Acta Crystallogr. Sect. D.

[66] Daura X., Gademann K., Jaun B., Seebach D., van Gunsteren W.F., Mark A.E. (1999). Peptide Folding: When Simulation Meets Experiment. Angew. Chem. Int. Ed..

[67] Frishman D., Argos P. (1995). Knowledge-based secondary structure assignment. Proteins Struct. Funct. Genet..

[68] Humphrey W., Dalke A., Schulten K. (1996). VMD—Visual Molecular Dynamics. J. Mol. Graph..

[69] Prendergast L., McClurg U.L., Hristova R., Berlinguer-Palmini R., Greener S., Veitch K., Hernandez I., Pasero P., Rico D., Higgins J.M.G. (2020). Resolution of R-loops by INO80 promotes DNA replication and maintains cancer cell proliferation and viability. Nat. Commun..

[70] Stirling D.R., Swain-Bowden M.J., Lucas A.M., Carpenter A.E., Cimini B.A., Goodman A. (2021). CellProfiler 4: Improvements in speed, utility and usability. BMC Bioinform..

[71] Danovski G., Dyankova T., Stoynov S. (2018). CellTool: An open source software combining bio-image analysis and mathematical modeling. bioRxiv.

[72] Schwab R.A., Niedzwiedz W. (2011). Visualization of DNA Replication in the Vertebrate Model System DT40 using the DNA Fiber Technique. J. Vis. Exp..

[73] Kirilov T., Gospodinov A., Kirilov K. (2023). An algorithm and application to efficiently analyse DNA fibre data. Biotechnol. Biotechnol. Equip..

