# Supplementary material for: Insights into the SARS-CoV-2 ORF6 Mechanism of Action
Source: Int J Mol Sci. 2023 Jul 18;24(14):11589. doi: 10.3390/ijms241411589 (PMC10380535; doi:10.3390/ijms241411589)
Supplement: Supplementary file 1 [file ijms-24-11589-s001.zip › ijms-2456746-supplementary.pdf]

Supplementary Materials: Insights into the SARS-CoV-2 ORF6 Mechanism of Action

Elena Krachmarova[1], Peicho Petkov[2,*], Elena Lilkova[3], Nevena Ilieva[3], Miroslav Rangelov[4], Nadezhda Todorova[5], Kristina Malinova[1], Rossitsa Hristova[1], Genoveva Nacheva[1], Anastas Gospodinov[1,*], Leandar Litov[2]

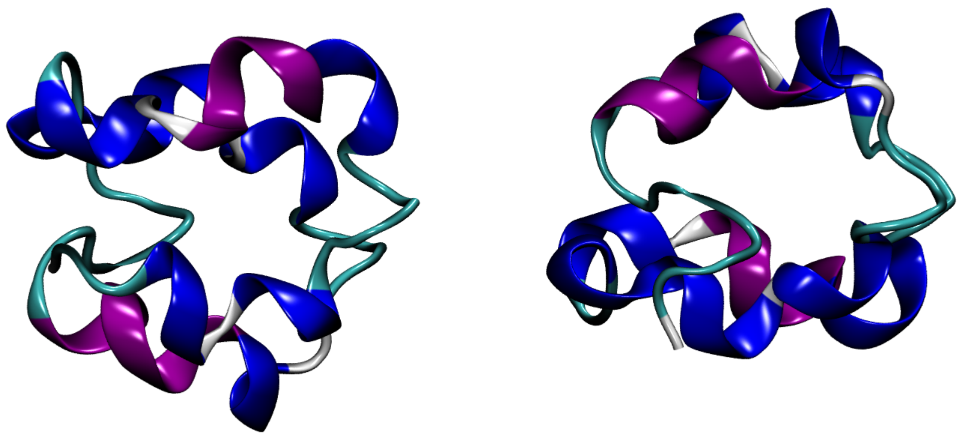

(a) **(b)**

Figure S1. Two different projections of the input model of ORF6 in water solvent, developed by the EXCALATE4COV project. The protein is coloured according to secondary structure, assigned by the STRIDE algorithm – α-helices are in purple, 3_{10}-helices – in blue, turns – in cyan and random coils – in white.

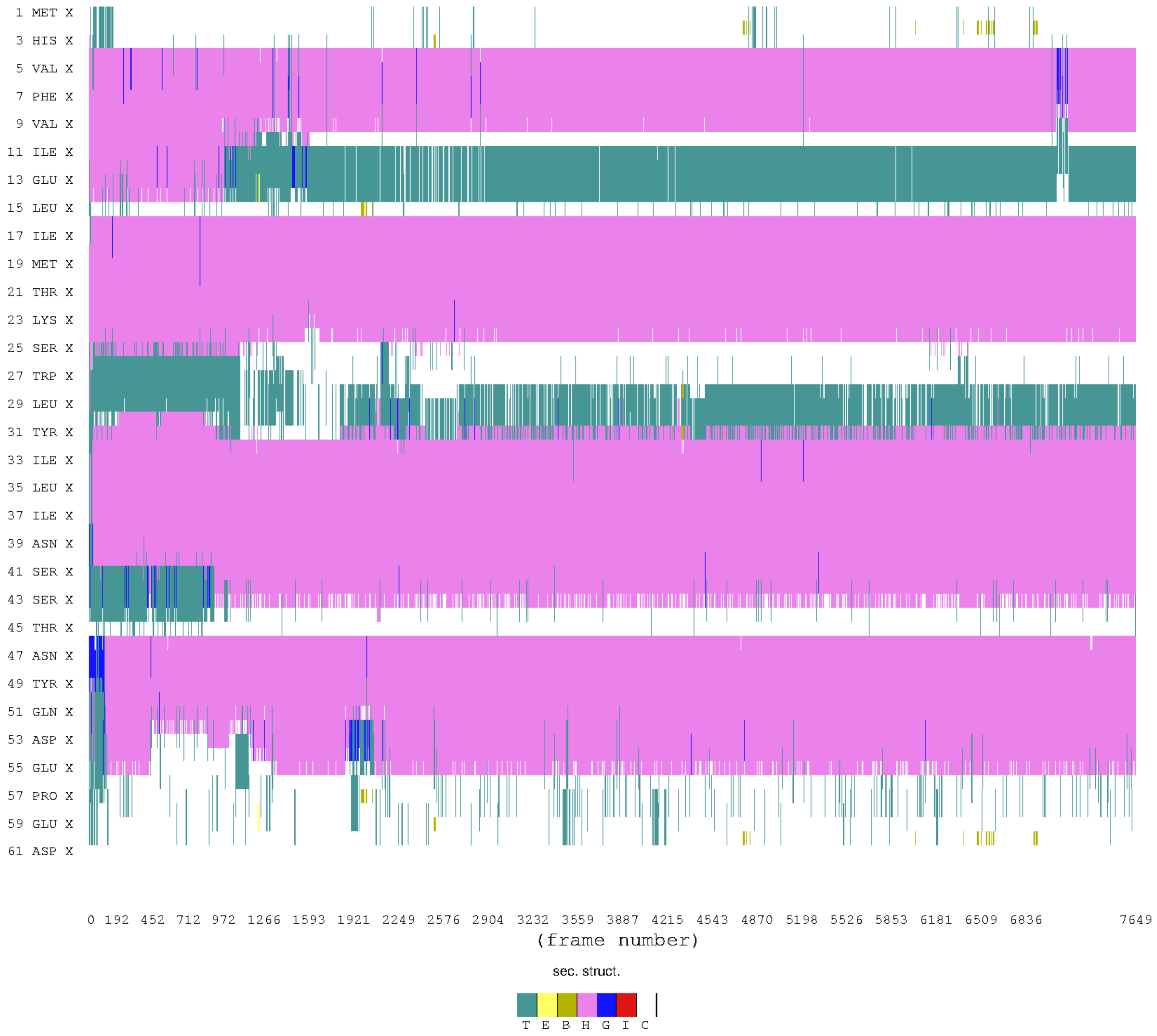

Figure S2. Secondary structure plot of the ORF6 in the folding simulation in water solvent. The secondary structure was assigned by the STRIDE algorithm – turns are shown in cyan, extended beta-structures – in yellow, beta-bridges – in ochre, α-helices – in purple, 3_{10}-helices – in blue, pi-helices – in blue and random coils – in white.

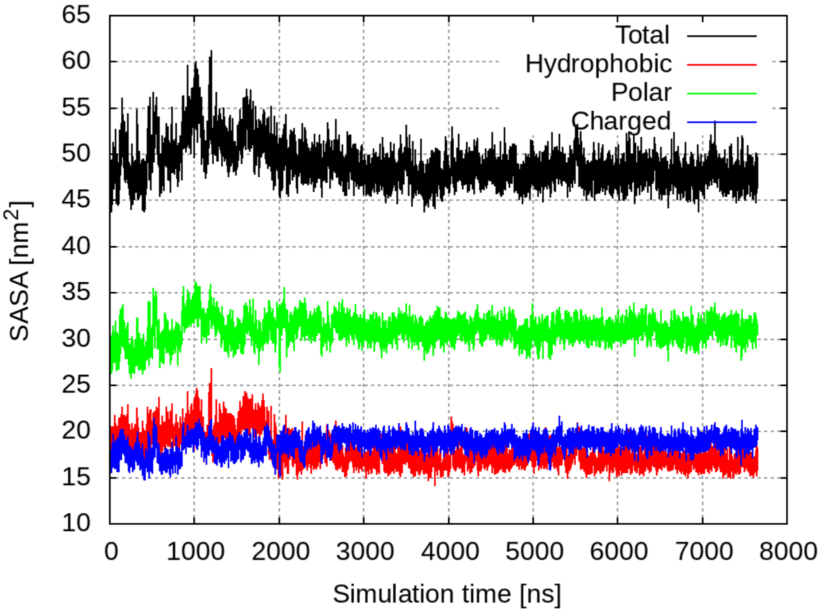

Figure S3. SASA plot of the ORF6 in the folding simulation in water solvent.

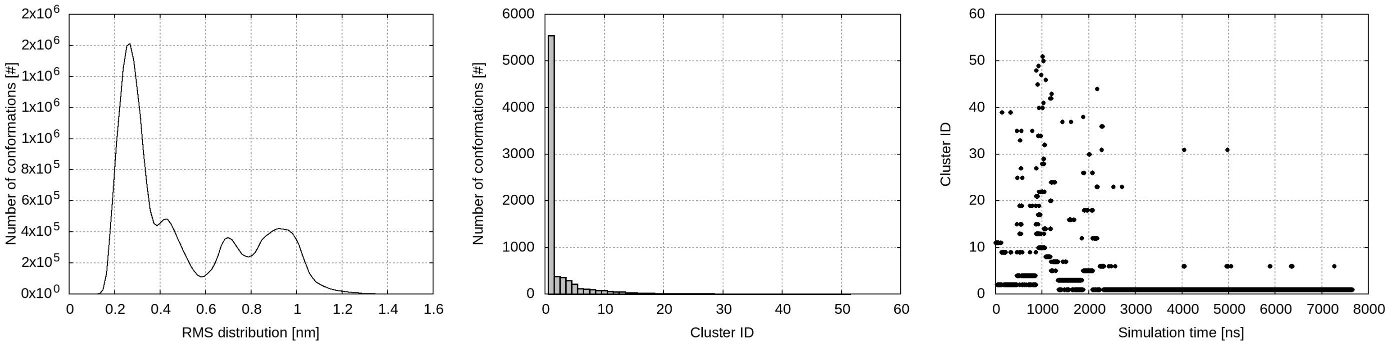

(a) **(b)** **(c)**

Figure S4. Cluster analysis of the ORF6 folding simulation in water solvent. (a) RMSD distribution of the conformations; (b) Cluster size; (c) Cluster occupancy.

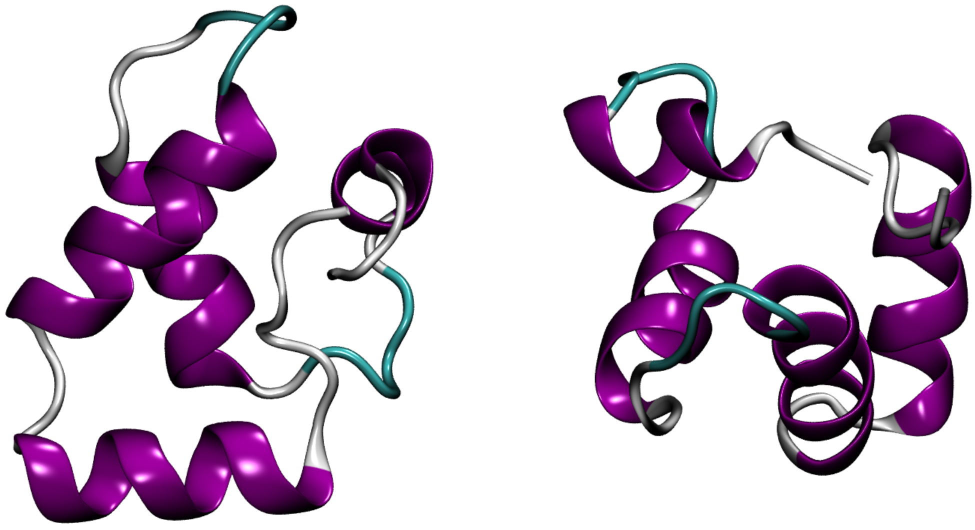

(a) **(b)**

Figure S5. Two different projections of the centroid of the largest cluster in the folding simulation of ORF6 in water solvent. The protein is coloured according to secondary structure, assigned by the STRIDE algorithm – α-helices are in purple, 3_{10}-helices – in blue, turns – in cyan and random coils – in white.

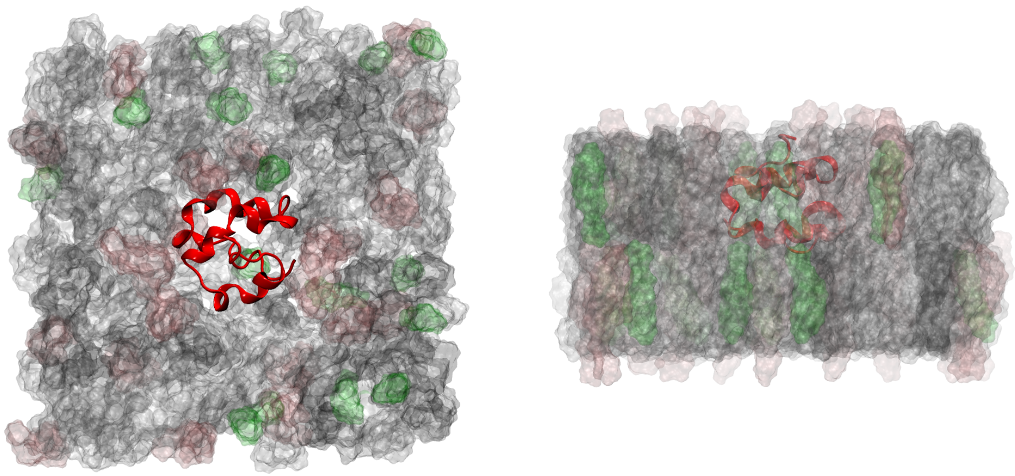

(a) **(b)**

Figure S6. Input model of ORF6 embedded in a model ER membrane. The protein is depicted in red cartoon representation, and the various lipid surfaces are coloured as follows: POPC – in white, POPE – in grey, POPI – in red, CHOL – in green.

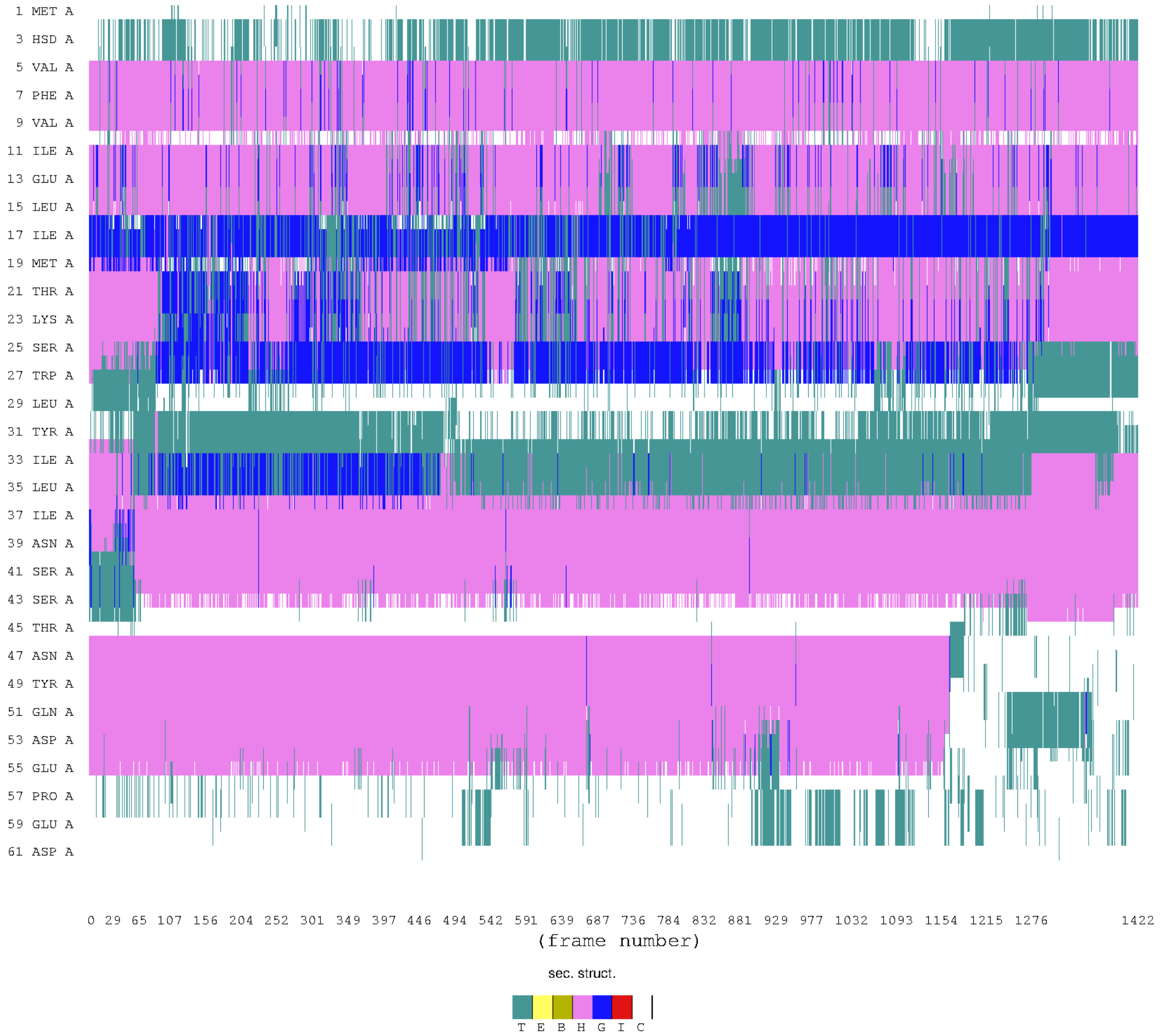

Figure S7. Secondary structure plot of ORF6 embedded in a model ER membrane. The secondary structure was assigned by the STRIDE algorithm – turns are shown in cyan, extended beta-structures – in yellow, beta-bridges – in ochre, α-helices – in purple, 3_{10}-helices – in blue, pi-helices – in blue and random coils – in white.

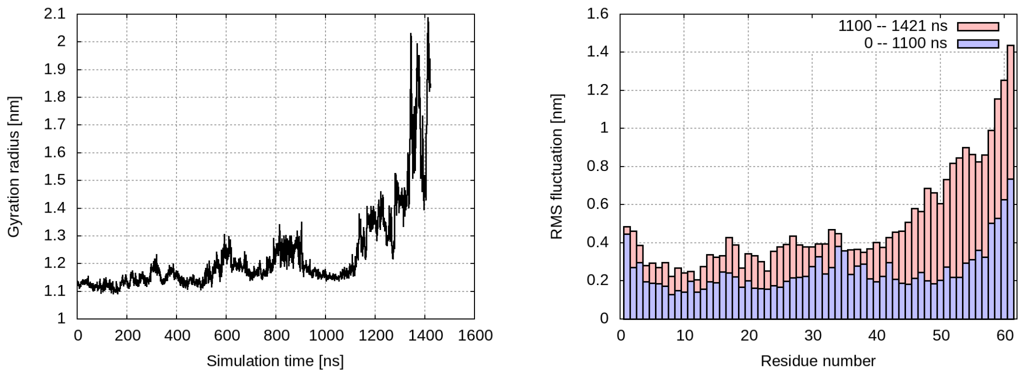

(a) **(b)**

Figure S8. (a) Gyration radius of the ORF6 molecule embedded in a model ER membrane as a function of the simulation time. (b) Root mean square fluctuations of the amino acid residues of ORF6 embedded in a model ER membrane. The blue boxes correspond to the RMSF calculated over the 0-1100 ns trajectory part, while the red boxes represent the trajectory part in which the C-terminal tail of the sticks out of the membrane and into the water solvent.

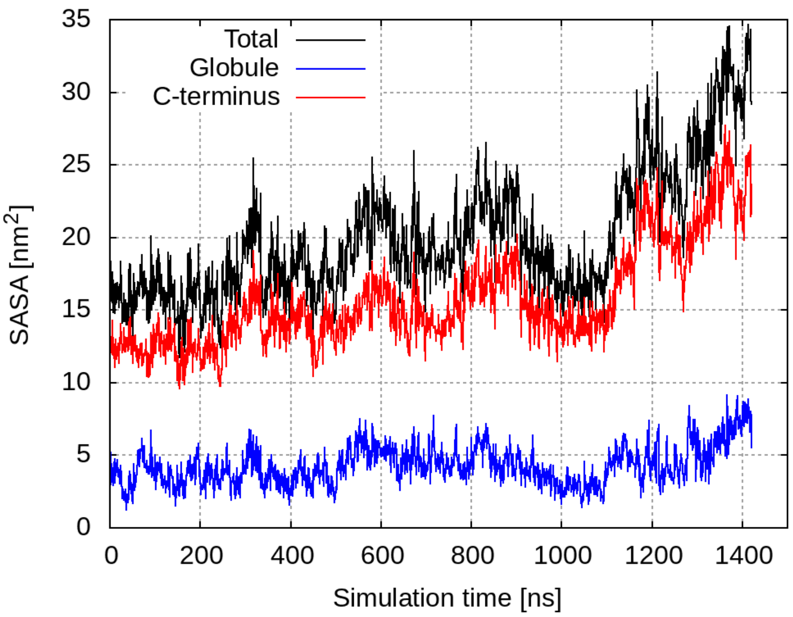

Figure S9. SASA of the ORF6 molecule embedded in a model ER membrane. The total SASA is shown in black, the SASA of the globular part of the protein (aa 1–40) is in blue and the SASA of the C-terminus (aa 41–61) is in red.

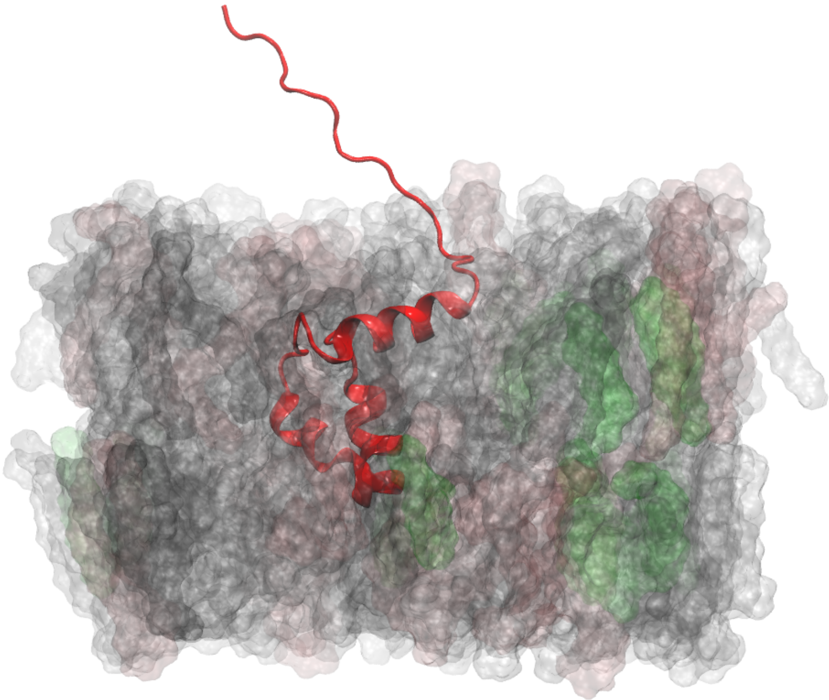

Figure S10. ORF6 as embedded in a model ER membrane. The protein is in red new cartoon representation, while the various lipid surfaces are coloured as follows: POPC – in while, POPE – in gray, POPI – in red, CHOL – in green. Stable conformation was achieved after 1.4 μs MD simulation.

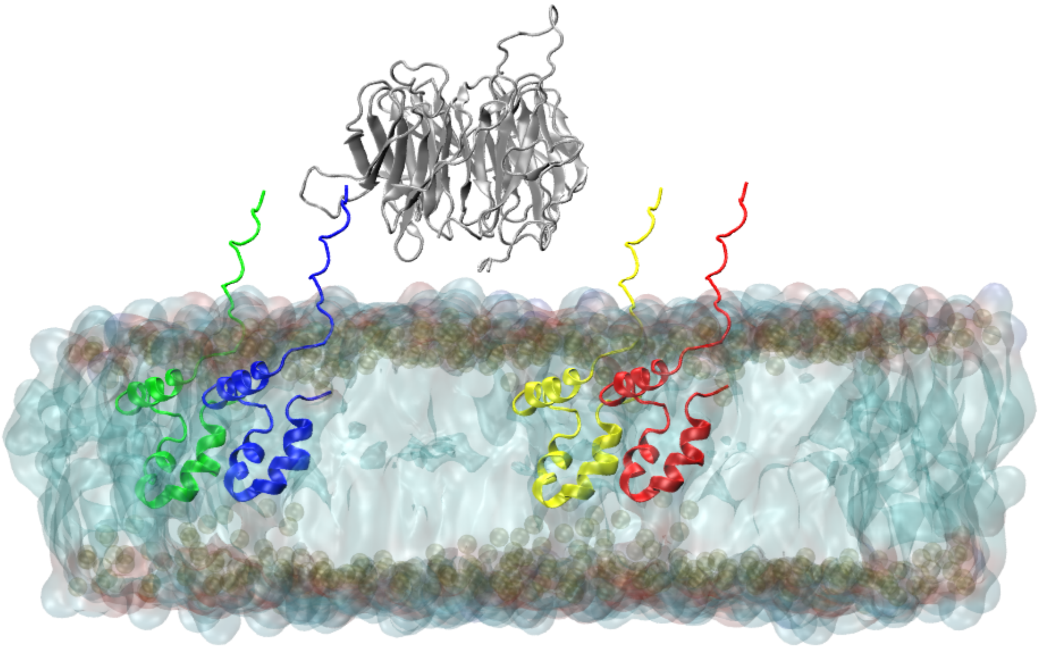

Figure S11. Input model of RAE1 (in grey cartoon representation) and four ORF6 molecules (in red, blue, green, and yellow cartoon representation) embedded in a model ER membrane.

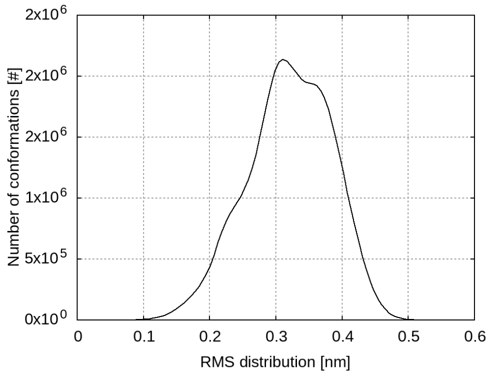

(a)

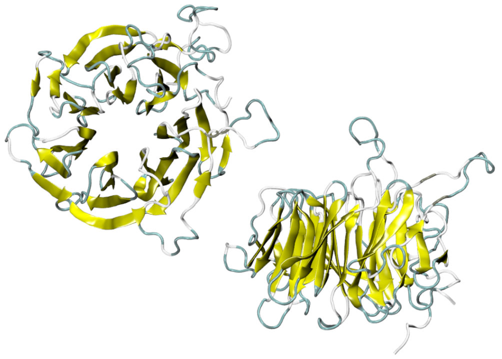

(b)

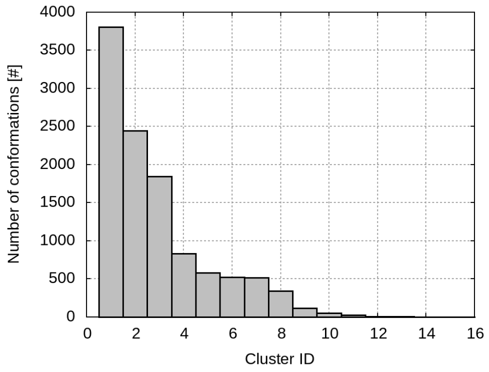

(c)

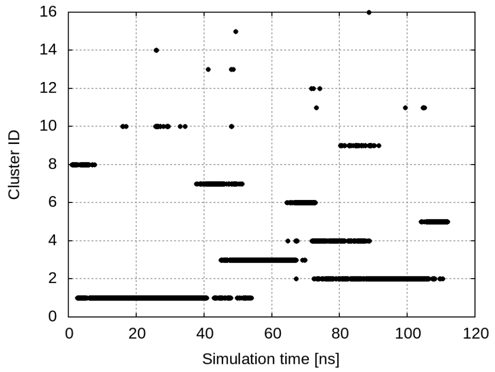

(d)

Figure S12. (a) Gyration radius of the ORF6 molecule embedded in a model ER membrane as a function of the simulation time. (b) Root mean square fluctuations of the amino acid residues of ORF6 embedded in a model ER membrane. The blue boxes correspond to the RMSF calculated over the 0-1100 ns trajectory part, while the red boxes represent the trajectory part in which the C-terminal tail of the sticks out of the membrane and into the water solvent.

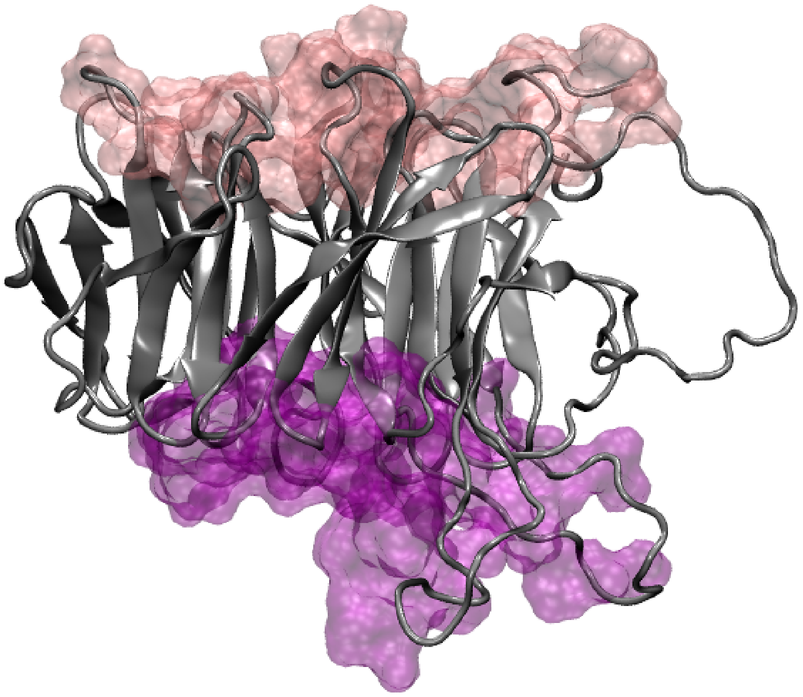

Figure S13. Comparison between the RAE1 (gray cartoon representation) binding interfaces of ORF6 molecule 2, represented in pink surface; and NUP98 – in purple surface.

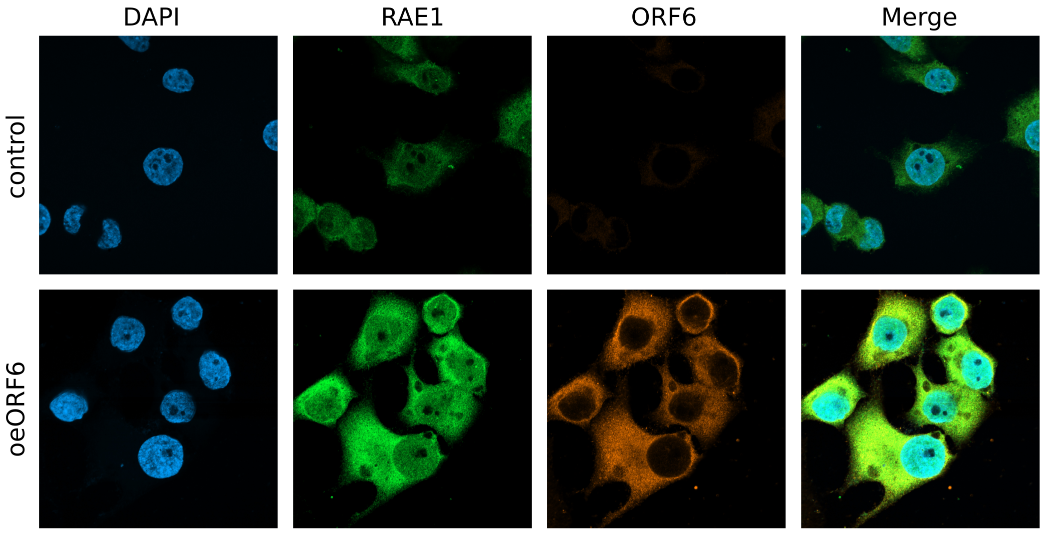

Figure S14. ORF6 co-localizes with Rae 1 and alters its subcellular localization. Confocal images of WISH cells transfected with the ORF6-expressing plasmid (oeORF6) and control cells (transfected with empty plasmid) stained with antibodies against RAE1 and ORF6. Representative images are shown.

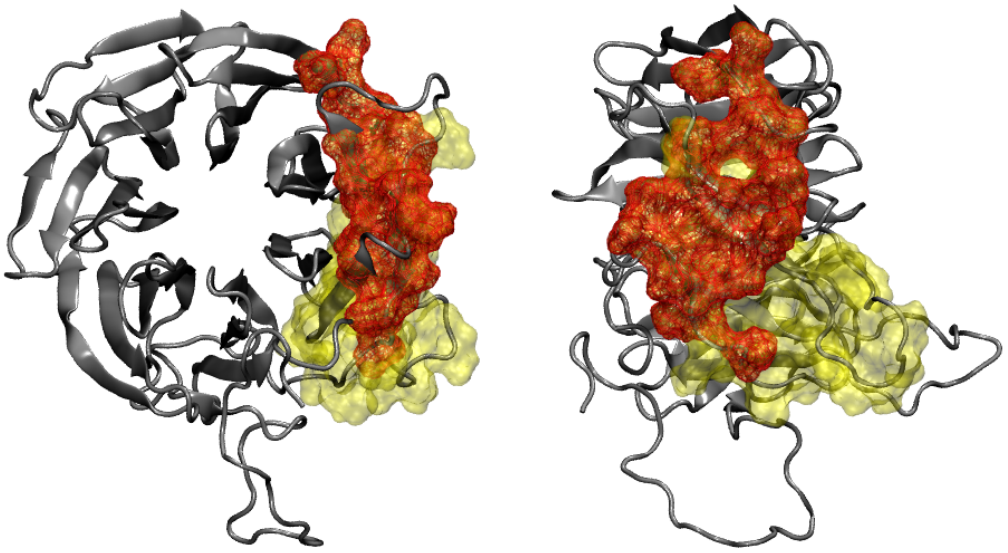

(a) **(b)**

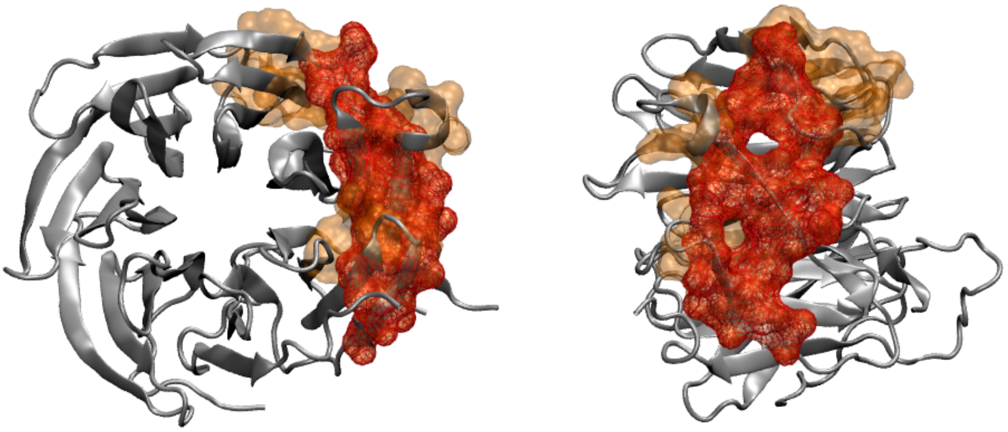

(c) **(d)**

Figure S15. (a) and (b) Comparison between the RAE1 (gray cartoon representation) binding interfaces of ORF6 molecule 1, represented in yellow surface; and (c) and (d) VSV M-protein – in orange surface. The shared interface is shown in red wireframe surface.

Table S1. List of Primers

Gene	Forward Primer	Reverse primer
RAE1	5′ GGG CTC CTG GGA TTT CTG TC 3′	5′ GGG CTC CTG GGA TTT CTG TC 3′
CCNE1	5′ GGT CCA CAG GGA TGC GAA G 3′	5′ CGA TTT TGG CCA TTT CTT CAT CTG G 3′
ACTB	5′ CAC CAT TGG CAA TGA GCG GTT C 3′	5′ AGG TCT TTG CGG ATG TCC ACG T 3′

Abbreviations

The following abbreviations are used in this manuscript:

ACTB	actin beta
CCNE1	cyclin E
CHOL	cholesterol
POPC	1-palmitoyl-2-oleoyl-sn-glycero-3-phosphocholine
POPE	1-palmitoyl-2-oleoyl-sn-glycero-3-phosphoethanolamine
POPI	1-palmitoyl-2-oleoyl-sn-glycero-3-phosphoinositol
RAE1	ribonucleic acid export 1
